# Adjuvant formulated virus-like particles expressing native-like forms of the Lassa virus envelope surface glycoprotein are immunogenic and induce antibodies with broadly neutralizing activity

**DOI:** 10.1038/s41541-020-00219-x

**Published:** 2020-08-04

**Authors:** Helena Müller, Sarah Katharina Fehling, Jens Dorna, Richard A. Urbanowicz, Lisa Oestereich, Yvonne Krebs, Larissa Kolesnikova, Martin Schauflinger, Verena Krähling, N’Faly Magassouba, Elisabeth Fichet-Calvet, Jonathan K. Ball, Andreas Kaufmann, Stefan Bauer, Stephan Becker, Veronika von Messling, Thomas Strecker

**Affiliations:** 1 Institute of Virology, Philipps University Marburg, Marburg, Germany; 2 Institute of Immunology, Philipps University Marburg, Marburg, Germany; 3 Wolfson Centre for Global Virus Infections, University of Nottingham, Nottingham, UK; 4 School of Life Sciences, University of Nottingham, Nottingham, UK; 5 Bernhard-Nocht Institute for Tropical Medicine, Hamburg, Germany; 6 German Center for Infection Research (DZIF), Partner Sites Gießen-Marburg-Langen and Hamburg-Borstel-Lübeck-Riems, Hamburg, Germany; 7 Veterinary Medicine Division, Paul-Ehrlich-Institut, Langen, Germany; 8 Laboratoire des Fièvres Hémorragiques Virales, Conakry, Guinea; 9 Present Address: Federal Ministry for Education and Research, Berlin, Germany

**Keywords:** Viral infection, Vaccines

## Abstract

*Lassa mammarenavirus* (LASV) is a rodent-borne arenavirus endemic to several West African countries. It is the causative agent of human Lassa fever, an acute viral hemorrhagic fever disease. To date, no therapeutics or vaccines against LASV have obtained regulatory approval. Polyclonal neutralizing antibodies derived from hyperimmunized animals may offer a useful strategy for prophylactic and therapeutic intervention to combat human LASV infections. The LASV envelope surface glycoprotein complex (GP) is the major target for neutralizing antibodies, and it is the main viral antigen used for the design of an LASV vaccine. Here, we assessed the immunogenic potential of mammalian cell-derived virus-like particles (VLPs) expressing GP from the prototypic LASV strain Josiah in a native-like conformation as the sole viral antigen. We demonstrate that an adjuvanted prime-boost immunization regimen with GP-derived VLPs elicited neutralizing antibody responses in rabbits, suggesting that effective antigenic epitopes of GP were displayed. Notably, these antibodies exhibited broad reactivity across five genetic lineages of LASV. VLP-based immunization strategies may represent a powerful approach for generating polyclonal sera containing cross-reactive neutralizing antibodies against LASV.

## Introduction

*Lassa mammarenavirus* (LASV), an Old World member of the family *Arenaviridae*, is the zoonotic agent of human Lassa fever (LF), which is endemic to several West African countries and affects an estimated 100,000–300,000 people annually^1,2^. While most cases of LF are asymptomatic or have mild symptoms, 20% of infections result in clinical manifestations varying from flu-like syndromes to fatal hemorrhagic fevers. The only drug with proven efficacy is the nucleoside analog ribavirin; however, treatment is only effective at an early stage of infection and is associated with adverse drug reactions^3^. Since no licensed therapeutic approaches or protective vaccines are yet available, LASV represents a severe public health problem in the affected regions. LF has been listed in the WHO R&D Blueprint of priority diseases requiring urgent research and development attention^4^. Ongoing large LF outbreaks associated with high case fatality rates in Nigeria underscore the critical need for accelerated research activities toward approved therapeutics and vaccines^5^. Furthermore, LASV is one of the most common viral hemorrhagic fever agents in risk group 4 exported to countries outside the endemic areas.

LASV is a lipid-enveloped virus that contains a single-stranded, bi-segmented RNA genome with an ambisense coding strategy. The envelope glycoprotein complex (GP) is exposed on the surface of the virus as trimeric spikes and is the main target for antibody-based therapeutics and vaccine design. Initially, the glycoprotein open reading frame (ORF) codes for an immature precursor polyprotein (referred to as GPC)^6^. A sequence of co- and posttranslational cleavage events by signal peptidase and the host cell convertase SKI-1/S1P generates the glycoprotein subunits stable signal peptide (SSP), GP1 and GP2, which remain associated through non-covalent interactions^7,8^. Proteolytic maturation is essential for GP function and virus infectivity^7^.

Fully assembled GP on the viral surface is displayed as a trimer of heterodimers composed of GP1 and GP2, which mediate binding to host cell receptors and virus–host membrane fusion, respectively^9–12^. A unique structural feature of GP from LASV and other arenaviruses is that SSP remains associated with the glycoprotein spike complex through interaction with GP2. SSP plays important roles in the maturation and assembly of GP as well as in the membrane fusion process required for virus entry (reviewed in ref. ^13^). In addition to SSP, the cytoplasmic domain of GP2 is a critical component for the stability and conformation of the external immunogenic GP domains^14^. Furthermore, the GP spike is extensively glycosylated, with ~30% of its mass consisting of host-derived N-linked glycans^15^. These N-linked glycans are essential for correct folding, cleavage, and transport of GP and also for efficient viral entry and infectivity^15–17^. The high density of glycans on the GP surface is thought to shield the underlying protein from efficient immune surveillance^18,19^. Despite this immunological escape mechanism, neutralizing antibodies (nAbs) can emerge in individuals who recovered from LF. However, it is widely accepted that T-cell responses play the central role in controlling acute LASV infection and are the driving force of clinical improvement since nAbs do not appear until the late stage of convalescence and are detected only in low-to-moderate titers^20,21^.

Several GP-based vaccine candidates against LASV have shown to be effective in preventing the fatal outcome of LF disease in preclinical animal testing. These include various approaches, such as live attenuated arenavirus vaccines (e.g., the natural reassortant ML29 or recombinant Mopeia virus)^22,23^, viral vector-based vaccines (e.g., modified *Vaccinia virus*, vesicular stomatitis virus (VSV), measles virus, rabies virus, and yellow fever virus)^24–29^, and DNA-based vaccines^30,31^. Both humoral and cell-mediated immune responses are assumed to contribute to vaccine-mediated protection; however, immune markers that correlate with protective immunity differ between vaccine platforms^32^. Notably, repeated immunization of nonhuman primates with purified LASV particles that have been inactivated by gamma irradiation failed to protect animals^33^. While the antibody response had the same magnitude as that found in humans who had recovered from LF, vaccine-induced antibodies exhibited no neutralizing activity. The lack of protection was also assumed to be associated with a limited ability of the inactivated LASV vaccine to elicit a robust cell-mediated immune response.

In addition to prophylactic vaccination, passive antibody-mediated immunotherapy has gained renewed interest for the treatment of LF. Convalescent plasma (CP) therapy has shown controversial results in clinical and preclinical settings. Early studies in humans and animal models demonstrated enhanced survival or even complete protection against LF following the administration of CP. Several factors were identified for effective LF therapy and treatment success, such as the time of CP administration post infection, the nAb concentration, and the antigenic matching of the CP preparation^20,21,34–36^. In contrast to these studies, a controlled clinical CP trial indicated that passive antibody transfer did not protect recipients from fatal LF^3^. Recently, monoclonal antibodies with high neutralizing capacity against GP derived from memory B cells of LF survivors have been shown to provide protection against LF in a nonhuman primate model, supporting the concept of passive antibody therapy. Moreover, treatment with these monoclonal antibodies resulted in complete survival even when administered at advanced stages of the disease, underscoring the beneficial effect of nAbs in the treatment of severe LF^37^.

Although protective monoclonal antibodies represent powerful therapeutic tools, the logistical and experimental procedures required for the generation of human B-cell-derived nAbs from convalescent donors are time and cost intensive. In addition, slight changes in the epitope structure may render a monoclonal antibody unable to recognize the target antigen. In light of the high genetic diversity of LASV, the possible emergence of antigenically distinct LASV strains with epidemic potential suggests a need for alternative strategies for the generation of rapidly available therapeutic antibodies with neutralizing capacity, particularly in the early phase of an outbreak when convalescent donor material is not yet available. Polyclonal antibodies that have high neutralizing activity against LASV and are derived from hyperimmunized animals could represent an alternative means of rapidly producing therapeutic antibodies for the treatment of LF, as has been described for Ebola virus^38–40^.

Efficient neutralization of LASV correlates with antibody binding to the functional forms of trimeric GP^41,42^. As discontinuous epitopes presented on structurally mature and properly assembled GP are the relevant targets for nAbs, the most desirable GP-based immunogen should closely mimic the native trimeric spike structure as it is displayed on the viral surface. Virus-like particles (VLPs) have been explored for a variety of viruses as a means of preserving the functional and structural native glycoprotein form and thus the antigenic conformation in their natural lipid membrane environment. Due to their ordered repetitive surface structures as well as their morphological similarity to authentic viruses, VLPs have been shown to stimulate host immune cells efficiently, eliciting both humoral and cellular immune responses. For example, vaccination with VLPs derived from the highly pathogenic filoviruses, Ebola and Marburg virus, has shown protection from lethal virus infection in various animal models^43–45^. In addition to their suitability as vaccine platforms, VLPs have been used to generate pathogen-specific neutralizing antisera with therapeutic potential for the treatment of viral infections^46^. We previously described that the sole expression of LASV GP in mammalian cells is sufficient to produce VLPs displaying GP in its trimeric form on the envelope surface that closely mimic the structure of the virion-associated spike^47–49^. In this proof-of-concept study, we assessed the immunogenicity of adjuvanted GP-derived VLPs in rabbits. We show that the GP VLPs elicited a nAb response against LASV, suggesting that critical epitopes of GP were displayed. Our data suggest that the use of GP-derived VLPs is a safe and practical strategy for the generation of neutralizing polyclonal antibodies against LASV.

## Results

### Production of LASV GP VLPs for use as an antigen

**Fig. 1:**
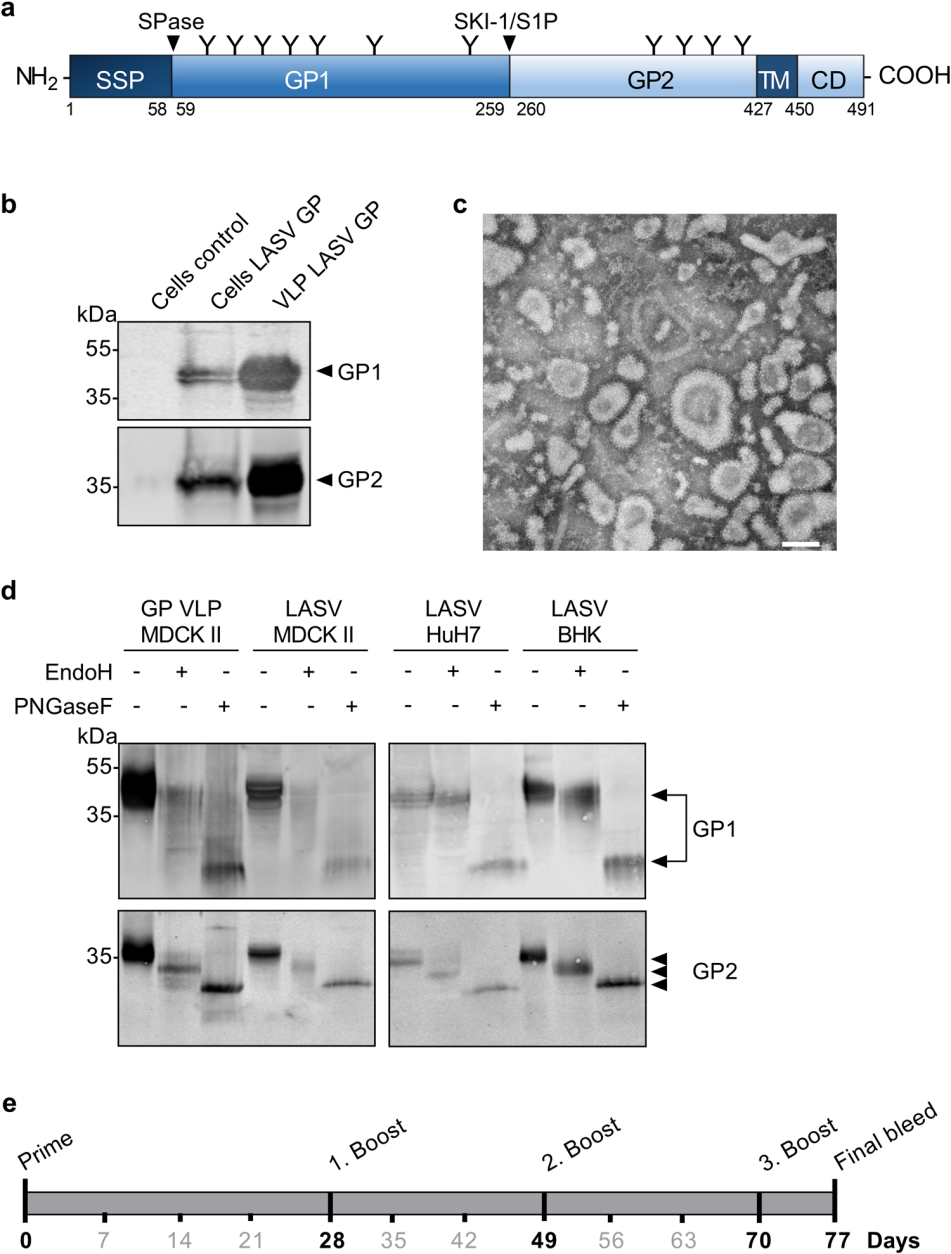
Production of LASV GP VLPs for use as immunogens. **a** Schematic representation of LASV GPC. The glycoprotein precursor GPC consists of the stable signal peptide (SSP) (amino acids 1–58), the receptor-binding subunit GP1 (amino acids 59–259), and the fusion-mediating subunit GP2 (amino acids 260–491) containing a transmembrane domain (TM) and a cytoplasmic domain (CD). The signal peptidase (SPase) cleavage site (after amino acid 58), the SKI-1/S1P cleavage site (after amino acid 259), and potential glycosylation sites (Y) are indicated. **b** Western blot analysis of LASV GP VLPs purified from the cell culture supernatant of MDCK II cells stably expressing LASV GP using primary antibodies against GP1 (mouse AC1) and GP2 (rabbit α4). **c** Electron micrograph of negatively stained, purified GP VLPs. Scale bar, 100 nm. **d** Western blot analysis of LASV GP after treatment with endoglycosidases. LASV GP VLPs released from MDCK II LASV GP cells and authentic LASV purified from infected MDCK II cells, HuH7 cells, or BHK cells were treated with either endo-β-N-acetylglucosaminidase H (EndoH) or N-glycosidase F (PNGaseF). GP subunits were detected with primary antibodies against GP1 (mouse AC1) and GP2 (rabbit α4), respectively. **e** Schematic diagram of the immunization protocol. New Zealand White rabbits were immunized intramuscularly with 300 μg VLPs mixed with Sigma Adjuvant and boosted 28, 49, and 70 days after the first immunization. Serum samples were taken at day 0 and immediately before each boost. Final blood collection was performed on day 77.

The GP of LASV mediates virus attachment and virus entry into human cells and is the sole target for the induction of nAbs. Due to their ability to display viral envelope glycoproteins in their native form, VLPs are suitable candidates for the induction of robust immune responses. Here, we aimed to assess the immunogenic potential of mammalian cell-derived VLPs that display LASV GP from the prototypic strain Josiah in its mature, fully glycosylated form (Fig. 1a). To facilitate large-scale production of the GP VLPs, we used our previously established Madin–Darby canine kidney strain II (MDCK II) cell line that stably expresses LASV GP and constitutively produces GP VLPs^47^. As shown in Fig. 1b, western blot analysis using an LASV GP1-specific monoclonal antibody and LASV GP2-specific polyclonal antibodies confirmed the presence of the GP1 and GP2 subunits in the VLPs purified from the cell culture supernatant by ultracentrifugation through a 20% sucrose cushion. Electron microscopic analysis of negative-stained VLPs revealed pleomorphic particles of different sizes that displayed GP spikes on their surfaces (Fig. 1c). As canine-derived MDCK II cells do not represent a natural host cell type for LASV, and recombinantly generated GP may not resemble native-like glycosylation of infectious LASV particles, we analyzed the glycosylation status of GP expressed on the surface of VLPs in comparison with GP present on authentic LASV virions purified from infected MDCK II cells. In addition, we assessed the glycosylation profile of GP from purified LASV virions released from infected human-derived HuH7 cells as well as Baby hamster kidney (BHK) cells that mimic virus shedding from rodents. Enzymatic deglycosylation assays using endo-β-N-acetylglucosaminidase H (EndoH) and PNGaseF digestion showed that the glycosylation profile of GP from both VLPs and authentic LASV secreted from MDCK II cells closely resembles that of naturally occurring LASV particles released from human and rodent cells. Recombinantly expressed GP on the surface of VLPs and GP on the surface of infectious LASV particles released from all mammalian cell lines tested were equally sensitive to endoglycosidase treatments and exhibited similar migration patterns in SDS-PAGE gels (Fig. 1d). The purified GP VLPs were subsequently used to determine their suitability as immunogens. Therefore, two New Zealand White rabbits were immunized intramuscularly with 300 μg VLPs mixed in a 1:1 ratio with squalene-containing water-in-oil Sigma Adjuvant, which was recently demonstrated to enhance the induction of functional antibody titers against Ebola virus and *Nipah virus*^46^. Both rabbits received three booster injections with the adjuvanted GP VLP preparation at day 28, day 49, and day 70, as depicted in the schematic representation of the immunization scheme (Fig. 1e). Blood samples were taken at day 0 and immediately before each boost. Final blood samples were collected on day 77. IgG antibody responses against LASV GP were assessed by immunofluorescence and immunoelectron microscopy analysis, immunoperoxidase monolayer assay (IPMA), and enzyme-linked immunosorbent assay (ELISA). Functional activity of antibodies was determined by antibody neutralization assays using authentic LASV and two surrogate systems for the study of neutralization under BSL2 containment conditions.

### Induction of LASV GP-specific antibodies in rabbits immunized with adjuvanted GP VLPs

**Fig. 2:**
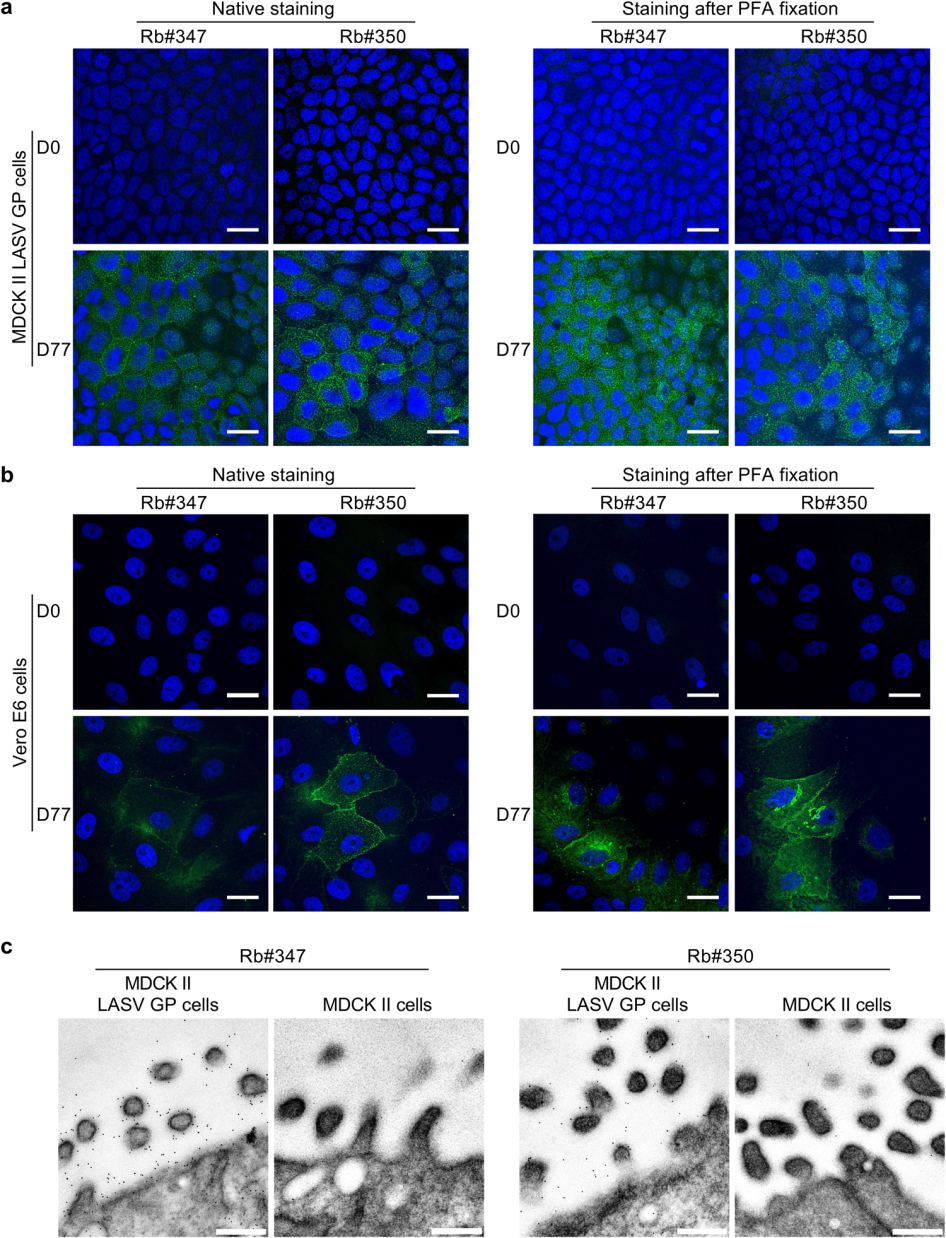
Reactivity of GP VLP-immunized rabbit sera against full-length LASV GP. Immunofluorescence microscopy of **a** MDCK II cells stably expressing LASV GP or **b** Vero E6 cells transiently transfected with plasmids encoding LASV GP. For the staining of native antigens, the cells were incubated with either the pre-immune rabbit sera (D0 samples) or rabbit immune sera after final bleed (D77 samples) before fixation with 4% PFA. For the staining of fixed antigens, the cells were treated with 4% PFA and permeabilized and then incubated with indicated rabbit sera. Anti-rabbit Alexa Fluor 488 was used as a secondary antibody, and nuclei were visualized by DAPI staining. Merged images of LASV GP and nuclear staining are shown. Scale bars, 20 μm. **c** Immuno-EM of stable transfected (MDCK II LASV GPC cells) and nontransfected MDCK II cells. For immunogold labeling of GP, the cells were aldehyde fixed, pelleted, and embedded in LR White, and ultrathin sections were incubated with rabbit serum (Rb#347_D77 or Rb#350_D77) diluted 1:20, followed by protein A conjugated to 6 nm colloidal gold. Scale bars, 250 μm.

First, we evaluated the immune sera of both rabbits taken at exsanguination on day 77 for their ability to recognize LASV GP by immunofluorescence microscopy. To this end, we used either MDCK II cells stably expressing GP or Vero E6 cells transiently expressing GP. The D0 pre-immune sera of both rabbits were used as controls to exclude nonspecific binding of the polyclonal sera. Figure 2a, b shows that both immune sera (Rb#347_D77 and Rb#350_D77) recognize GP in its native conformation in live MDCK II and Vero E6 cells. In contrast, cells stained with the pre-immune sera did not show evidence of nonspecific antibody binding. As some of the below-mentioned serological techniques for measuring LASV-specific binding antibodies require chemical fixation, we wanted to assess whether paraformaldehyde (PFA) fixation of cells expressing GP interferes with antibody binding and antigen recognition by the rabbit immune sera due to potential PFA-induced structural changes and/or cross-linking of the GP antigen. However, staining of PFA-treated GP-expressing MDCK II and GP-transfected Vero E6 cells with both immune sera showed a similar staining pattern when compared with the staining of GP under native conditions, suggesting the preservation of antibody recognition sites within GP after chemical fixation. In addition, we performed immunoelectron microscopic analysis on ultrathin sections using the sera samples Rb#347_D77 and Rb#350_D77. Both rabbit sera recognize GP at the plasma membrane of the MDCK II LASV GP cells, while only a few gold particles were detected in untransfected MDCK II control cells (Fig. 2c). Collectively, our findings indicate that the expression of fully assembled GP antigen by recombinant VLPs induced LASV GP-specific binding antibodies.

### Quantification and characterization of LASV GP-specific antibodies

**Fig. 3:**
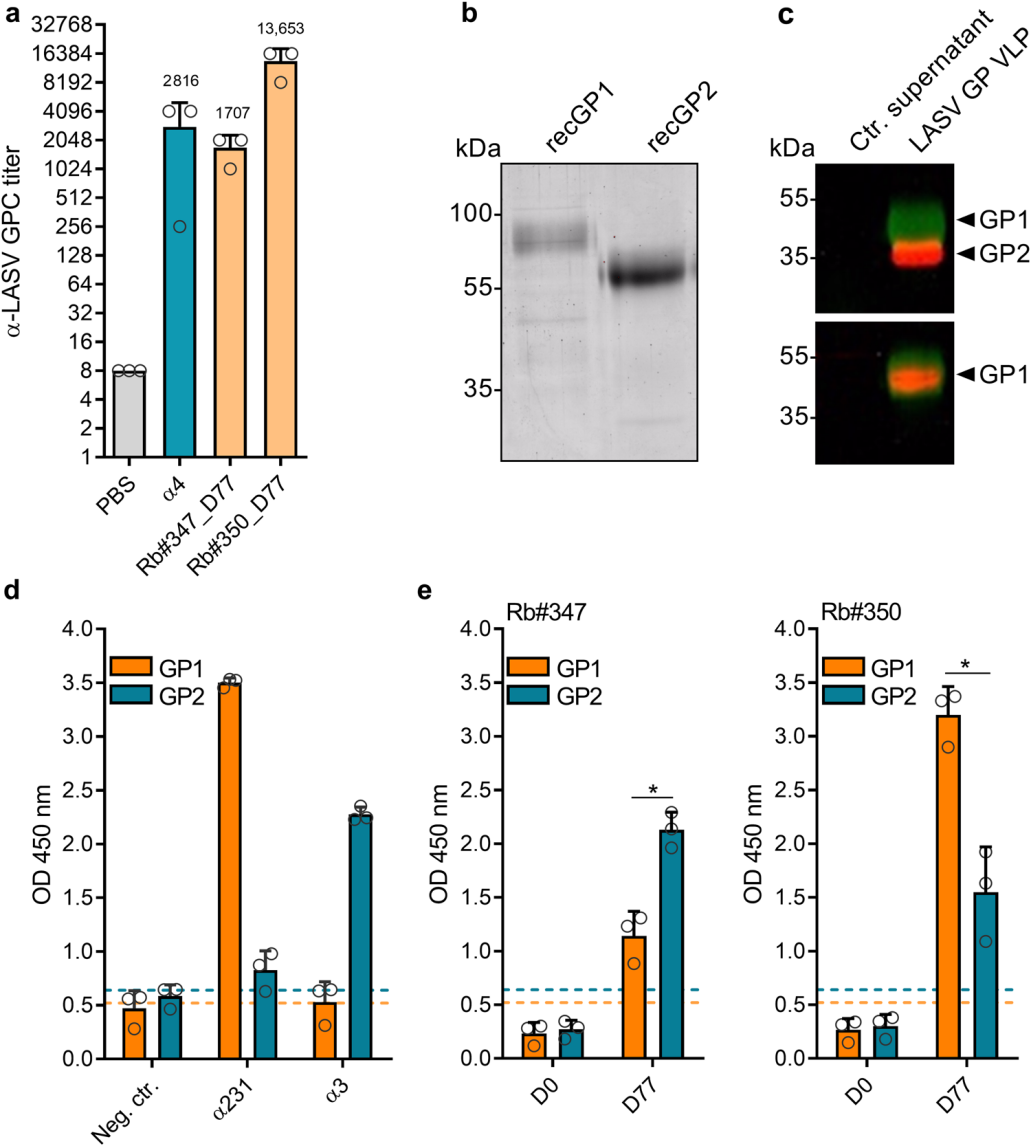
Quantification of LASV GP-specific antibodies. **a** IPMAs for total IgG against LASV GP of rabbit immune sera after final bleed (D77 samples) and control antibody (α4) are shown. The initial dilution was 1:16, and the total α-LASV GP titer was calculated as the reciprocal value of the last serum dilution at which positive staining was detected. Error bars are representative of the standard deviation (SD) and are calculated from three independent experiments. **b** SDS-PAGE and SYPRO Ruby protein gel staining analyses of recombinant LASV GP1 and GP2 domains that were used for ELISA plate coating. Estimated molecular masses are indicated. **c** Western blot analysis to confirm the specificity of rabbit control sera (α231 and α3) used for ELISA. Purified GP VLPs and control cell culture supernatant were separated by SDS-PAGE and blotted to nitrocellulose membranes. The upper blot was stained with mouse AC1 (against GP1) and rabbit α3 (against GP2), while the lower blot was stained with mouse AC1 and rabbit α231 (against GP1). **d** ELISA of total IgG against LASV GP1 or GP2 using rabbit control sera α231 and α3 diluted 1:200 as well as unrelated negative control sera. **e** ELISA using 1:200 dilution of sera from vaccinated animals (D0 and D77 samples). Error bars are representative of the SD of OD 450 nm values (duplicate measurement of three independent experiments). The dashed line depicts the cut-off for a positive antibody response, calculated as the median of the negative control + 10%. Statistical significance was calculated using an unpaired *t*-test (*p value < 0.05).

We then used IPMA to assess the level of the total IgG response of the rabbit immune sera against LASV GP. Polyclonal antibody α4 was used as a positive control. As shown in Fig. 3a, serum Rb#347_D77 exhibited a GP-specific antibody titer of 1:1707, while serum Rb#350_D77 had an eightfold higher final titer of 1:13,653. These results show that the LASV GP VLP immunization approach is immunogenic. It was then of interest to measure the specific antibody binding responses of the immune sera to either of the GP1 or GP2 subunits. Therefore, we developed an ELISA using recombinant LASV GP1 comprising amino acids 59–259 and recombinant LASV GP2 comprising amino acids 260–426 expressed in HEK293 cells. The purity of the recombinant GP1 and GP2 protein samples was confirmed by SYPRO Ruby protein gel staining (Fig. 3b). Of note, the presence of a human IgG Fc-tag and a glycine–serine linker in the recombinant proteins accounts for the higher molecular weight of GP1 and GP2 in the SDS-PAGE analysis. As controls for the GP1 and GP2-specific IgG ELISA, we used our in-house developed LASV GP-specific polyclonal rabbit sera designated α231 (recognizing LASV GP1) and α3 (recognizing LASV GP2). The specificity of both sera was confirmed by western blot analysis using purified LASV GP VLP preparations (Fig. 3c). ELISA results demonstrate the specific detection of recombinant GP1 and GP2 by the control sera α231 and α3, respectively, as indicated by the enhanced optical density (OD) 450 nm value for the corresponding GP subunit (Fig. 3d). Unrelated rabbit control serum was used as a negative control for the assay. When we assessed the serum reactivity from immunized animals toward either GP1 or GP2, we found that rabbit Rb#347 produced significantly more binding antibodies against the GP2 subunit in comparison with the level of binding antibodies against GP1, 77 days after the initial immunization. Conversely, animal Rb#350 generated significantly higher levels of binding antibodies directed to GP1 compared with the levels of binding antibodies specific to GP2 (Fig. 3e). No unspecific binding of both pre-immune sera (D0 samples) from the vaccinated animals was observed. These data demonstrate that VLP-based immunization co-formulated with a squalene-based adjuvant elicited antibodies targeting both GP subunits.

### Cross-reaction of polyclonal antibodies to GP of other LASV lineages and related Old World *mammarenaviruses*

**Fig. 4:**
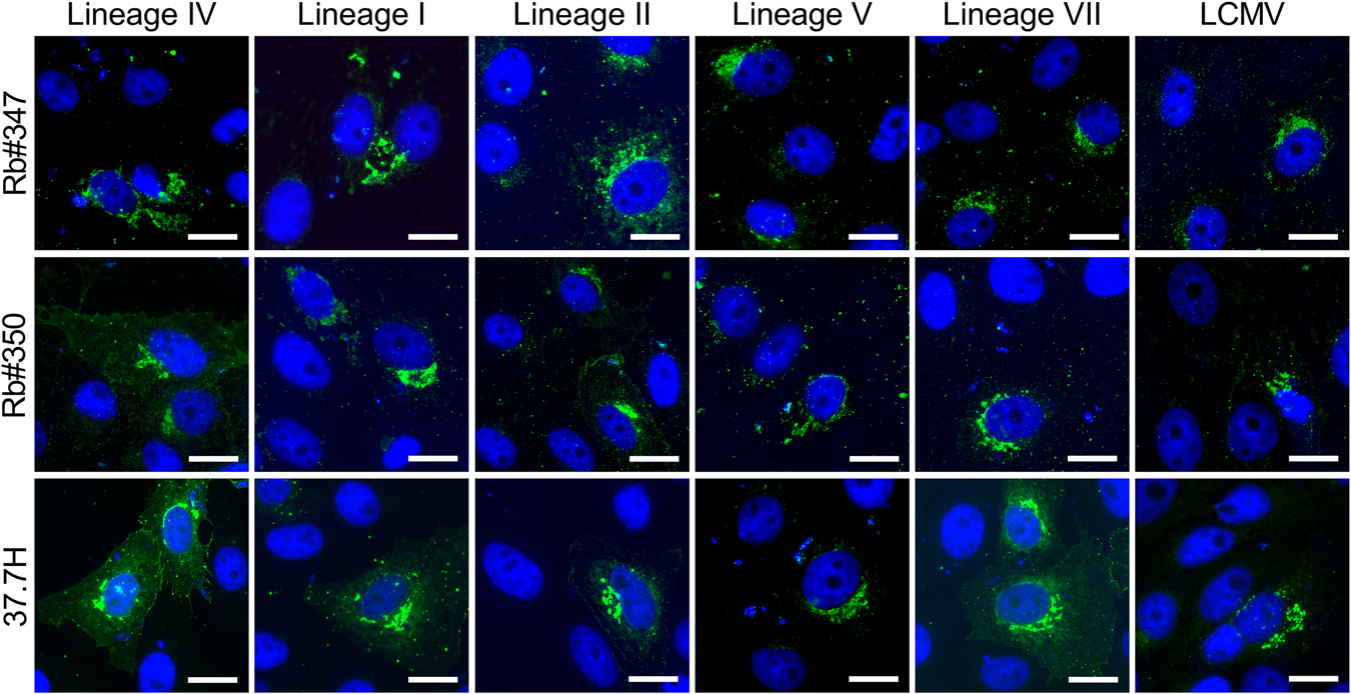
Antibody cross‐reactivity among LASV lineages and related Old World *mammarenaviruses*. GP of the LASV lineages I, II, IV, V, and VII or LCMV were vectorially expressed in Vero E6 cells. After fixation, cell monolayers were incubated with the rabbit immune sera Rb#347_D77 and Rb#350_D77, or, as a control, the human monoclonal antibody 37.7H, and analyzed by indirect immunofluorescence microscopy. Cell nuclei were visualized with DAPI, and merged images are shown. Scale bars, 20 µm.

We then determined if the polyclonal antibodies induced by immunization of VLPs expressing GP of the lineage IV Josiah strain have the potential to cross-react with GP from other LASV lineages and the related Old World *mammarenavirus* lymphocytic choriomeningitis virus (LCMV). Cross-reactivity was assessed by immunofluorescence microscopy using Vero E6 cells transiently expressing GP of the LASV lineages I, II, IV, V, and VII, or LCMV (Fig. 4). The broadly reactive human monoclonal antibody 37.7H served as a control^41^. Besides GP of LASV lineage IV, both rabbit immune sera effectively recognized the GP of all other LASV lineages tested. Both sera were also capable of binding to the GP of LCMV. The high degree of cross-reactive binding properties of the rabbit immune sera across multiple lineages of LASV and related Old World arenaviruses is in agreement with previous observations, demonstrating broad cross-reactivity of monoclonal anti-GP antibodies isolated from LF survivors^41^.

### nAb responses elicited by LASV GP VLP immunization

**Fig. 5:**
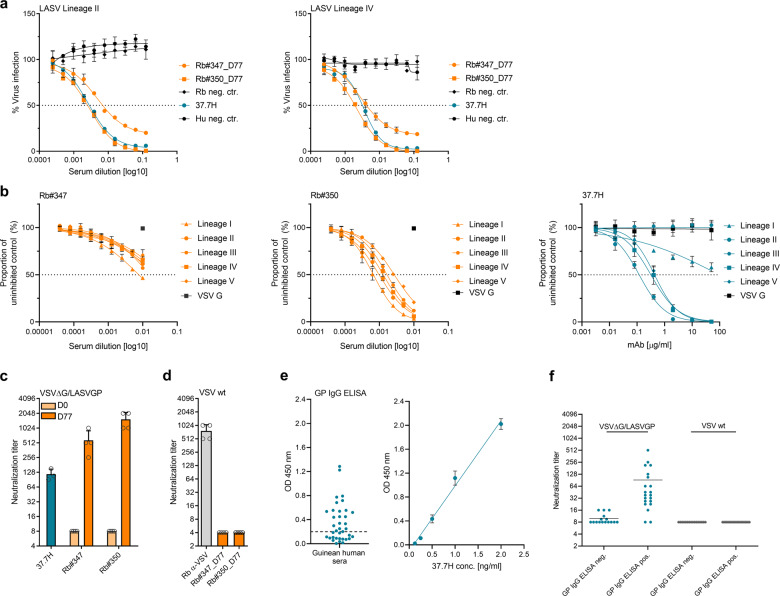
Neutralizing antibody responses elicited by LASV GP VLP immunization. **a** Serial dilutions of day 77 sera from VLP-immunized rabbits were incubated with either LASV lineage II (LASV/NGA/2018/IRR/015) or LASV lineage IV (Josiah), and Vero 76 cells were infected with the serum–virus mixture. An unrelated rabbit- or human-derived serum served as negative control, while monoclonal antibody 37.7H served as positive control. Infected cells were stained with a specific LASV NP antibody and an HRP-labeled secondary antibody. Mean values of neutralization titer [IC_50_ values] are plotted with error bars indicating standard deviations. **b** Breadth of neutralization against five lineages of LASV. Pseudoviruses generated with clones derived from five LASV lineages and VSV G were assessed for their neutralization sensitivity (50% neutralization titer [ID_50_]) to twofold serial dilutions of day 77 sera from Rb#347 and Rb#350, and 37.7H antibody as positive control. Mean values are plotted with error bars indicating standard deviations. **c** Serial dilutions of day 77 sera from immunized rabbits, and 37.7H control antibody were incubated with VSVΔG/LASVGP. Virus neutralization titers were calculated as geometric mean titers (GMT) of the reciprocal value of the last serum dilution at which inhibition of the cytopathic effect on infected Vero E6 cells was detectable. Pre-immune sera (D0 samples) from corresponding rabbits were used as controls. The initial dilution was 1:16 and was therefore denoted as a titer of eight for GMT calculation. Graphs represent the means from three independent experiments. Error bars indicate standard deviations. **d** Infectivity of wild-type VSV was determined in the presence of D77 serum samples. Polyclonal neutralizing antibodies against VSV were used as a positive control. **e** LASV GP IgG ELISA. A total of 37 human serum samples collected in an LASV-endemic area of Guinea were analyzed at 1:200 dilution for the detection of IgG antibodies against LASV GP. The mean of OD 450 nm values (duplicate measurement) are shown. The dashed line depicts the cut-off for a positive antibody response, calculated as the mean of human negative sera + twofold SD. A standard curve was prepared using the human monoclonal antibody 37.7H. Graphs represent the means from four independent experiments. Error bars indicate standard deviations. **f** Human sera were tested for neutralizing antibodies against LASV GP using VSVΔG/LASVGP. VSV expressing wild-type glycoprotein G was used as a control. Virus neutralization titers were calculated as GMT and the mean value of all measurements is shown.

The sera from VLP-immunized rabbits were then tested for their capacity to neutralize the infection of authentic LASV by immunofocus reduction neutralization assay. As shown in Fig. 5a, the Rb#347_D77 and Rb#350_D77 sera showed potent neutralizing activities against their homologous LASV strain Josiah (with IC_50_ values of 1:354 and 1:449, respectively). Furthermore, both rabbit immune sera cross-neutralized the infection by a clinical LASV isolate from lineage II^50^, although with slightly weaker activity (with IC_50_ values of 1:210 and 1:350, respectively). A rabbit- or human-derived negative control serum exhibited no neutralization activity against both tested viruses even at the lowest serum dilution. The monoclonal antibody 37.7H served as a positive control. Our results indicate that the VLP immunization elicited cross-nAb response in rabbits.

### Broadly neutralizing activity of hyperimmune sera

As we have shown that the VLP-immunized animals have a strong nAb response to LASV representatives of lineages II and IV, we decided to investigate if the nAbs elicited were capable of neutralizing additional LASV lineages. Using a retroviral pseudotype system as a BSL2 surrogate model of neutralization, we produced LASV pseudoviruses (LASVpv) expressing different LASV GPs representing five different lineages. Glycoprotein G from VSV was used as a negative control. As shown in Fig. 5b, D77 sera from both animals were capable of neutralizing all five LASV lineages, but neither serum was able to neutralize VSV G expressing pseudoviruses at the highest concentration of sera (1:100), showing that the neutralization measured was GP specific. Interestingly, lineage I was the most easily neutralized by sera from both animals (Rb#347, ID_50_ value of 1:130; Rb#350, ID_50_ value of 1:1507) rather than the homologous Josiah strain. Lineage V was the most refractive to neutralization, but was still susceptible (Rb#347, ID_50_ value of 1:25; Rb#350, ID_50_ value of 1:373). The serum from Rb#350 was more potent than that of Rb#347, with ID_50_ values ranging from 1:373 to 1:1507, which is reflective of what was seen in the other assays. Rb#350 was also able to neutralize lineage I (ID_50_ value of 1:1507) and lineage II (ID_50_ value of 1:1028) better than lineage IV (ID_50_ value of 1:781). Consistent with previous results, the monoclonal antibody 37.7H used as a positive control effectively neutralized the GPs from LASV lineage II and IV, but exhibited a reduced neutralizing activity toward GP from LASV lineage I. However, 37.7H failed to neutralize GP from lineage III, which contrasts previous observations^41^. The different neutralizing responses measured in the different assays may stem from different type of assay formats, which require further investigations. Altogether, these data show that the nAbs elicited in VLP-immunized animals are broadly neutralizing, as they inhibit entry of five different LASV lineages.

### Comparison of neutralizing activities

We then measured the level of nAb activity using replication-competent VSVΔG/LASVGP in which the original VSV glycoprotein G was replaced by LASV GP as another BSL2 surrogate system^51^. As shown in Fig. 5c, D77 sera from the VLP-immunized animals showed potent neutralizing activities against VSVΔG/LASVGP, with mean titers of 1:768 for Rb#347 and 1:2048 for Rb#350. In contrast, the pre-immune control sera (D0 samples) did not exhibit any neutralization activity at the lowest dilution tested (1:16) and were therefore denoted as a titer of eight for mean titer calculation. Monoclonal antibody 37.7H was used as a positive control (mean titer of 1:117). Both immune sera showed no neutralizing activity toward wild-type VSV, suggesting that the neutralizing capacity is specific to LASV GP (Fig. 5d). We used a guinea pig VSV-polyclonal antiserum as a control for efficient wild-type VSV neutralization. To estimate the power of neutralization by VLP immunization, we compared the neutralization titers of immunized animals with neutralization titers of human serum samples collected in a highly LASV-endemic area of Guinea (Fichet-Calvet and Magassouba, unpublished data). IgG antibodies specific for LASV GP were determined using a LASV GP ELISA (Fig. 5e). From a total 37 samples, 21 samples were reactive against LASV GP, while 16 samples were LASV GP seronegative. We then tested these samples for their neutralizing activities. Mean neutralization titers for all LASV GP seronegative sera were 1:10. The GP seropositive sera neutralized VSVΔG/LASVGP infection with a mean titer of 1:92 (Fig. 5e). No unspecific neutralizing effects were observed using wild-type VSV. These results indicate that the GP VLP immunization was superior in eliciting nAb responses compared with human serum samples that are IgG-positive for antibodies to LASV GP. Overall, these results show that the VLPs were capable of inducing GP-specific antibodies that effectively neutralize LASV.

### Induction of nAb titers requires several immunizations

**Fig. 6:**
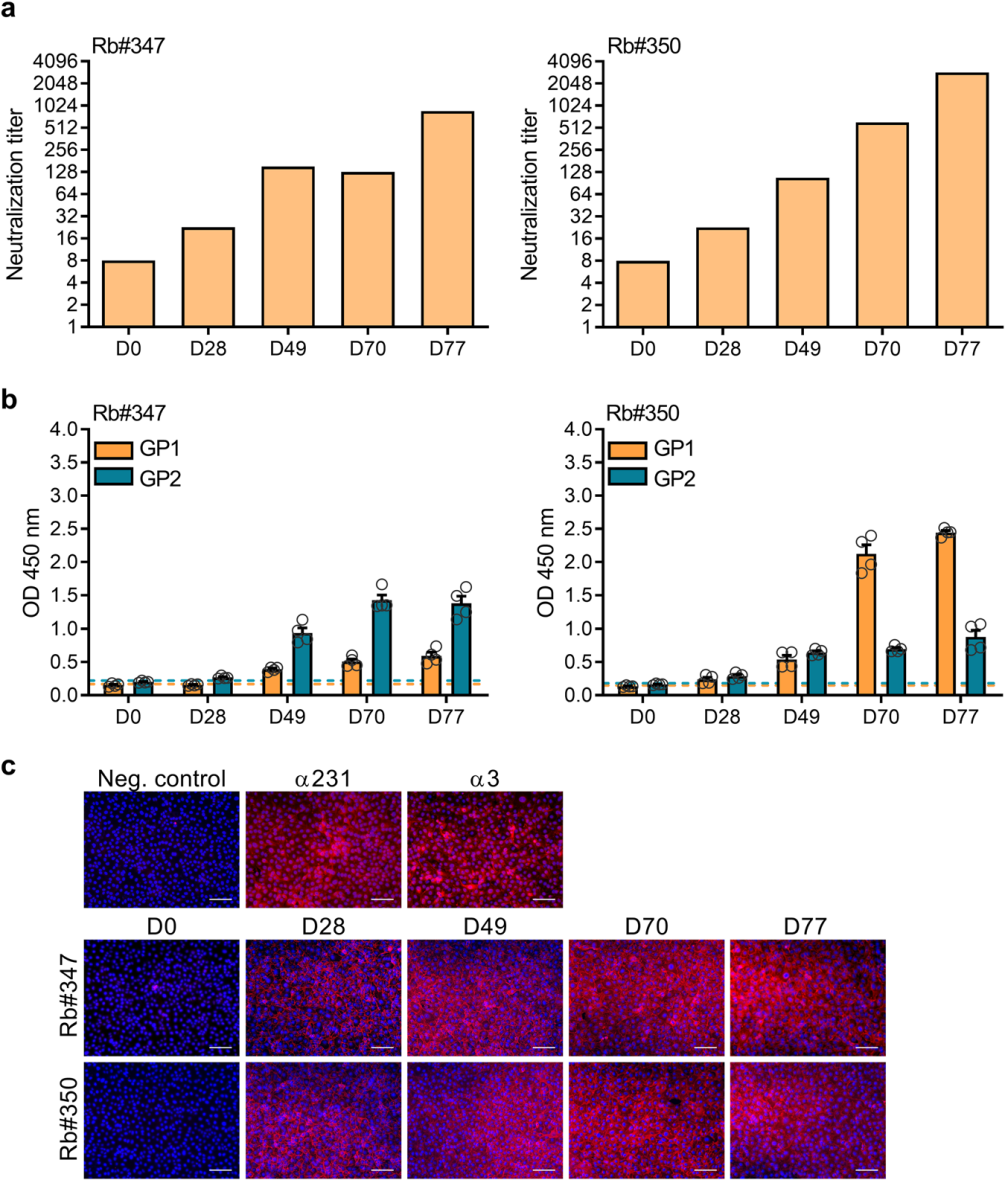
Induction of robust neutralizing antibody titers requires several immunizations. **a** Geometric mean neutralizing antibody titers against VSVΔG/LASVGP at 0, 28, 49, 70, and 77 days are shown (*n* = 1). The initial dilution was 1:16 and was therefore denoted as a titer of eight for GMT calculation. **b** ELISA of total IgG response against LASV GP1 or GP2 of 1:200 dilution of rabbit sera collected at indicated time points is shown. Error bars are representative of the SD of OD 450 nm values (duplicate measurement of two independent experiments). The cut-off for a positive antibody response is indicated as a dashed line, calculated as the median of the pre-immune sera (D0) + 10%. **c** Immunofluorescence microscopy using MDCK II cells stably expressing LASV GP demonstrates the recognition of GP by serum antibodies collected from immunized rabbits at indicated time points post-priming. An unrelated rabbit serum was used as a negative control, while α231 and α3 were used as a positive control. All sera were diluted 1:200. Anti-rabbit Alexa Fluor 568 was used as a secondary antibody, and nuclei were visualized by DAPI staining. Merged images of GP and nuclear staining are shown. Scale bars, 200 µm.

We next wanted to determine the kinetics of nAb development against LASV GP. Therefore, we assessed the neutralizing activity of sera collected at day 0, day 28, day 49, day 70, and day 77 of the prime-boost immunization regimen. Both animals developed low levels of nAb activity 4 weeks after priming, while repeated boosting substantially increased nAb titers (Fig. 6a). These data illustrate that repeated immunization of GP is required for efficient induction of functional nAbs. The increase in neutralizing activity correlated with the IgG ELISA results using recombinantly expressed GP1 and GP2 proteins, showing a continuous increase of GP-binding antibodies over the course of immunization (Fig. 6b). In addition, we performed immunofluorescence microscopy analysis using the stably transfected LASV GP MDCK II cell line to test whether the rabbit immune sera collected at the early time points post-priming recognize GP molecules expressed on the cell surface. GP expression was confirmed using the positive control sera α231 and α3. In contrast, cells that were immunostained with a negative control serum or the pre-immune sera (D0 samples) from immunized animals did not show any GP-specific signals. Consistent with our neutralization and ELISA data, all tested immune sera, including those from the early time points post-priming, have antibodies capable of recognizing GP (Fig. 6c).

### Mechanism of neutralization by polyclonal nAbs

**Fig. 7:**
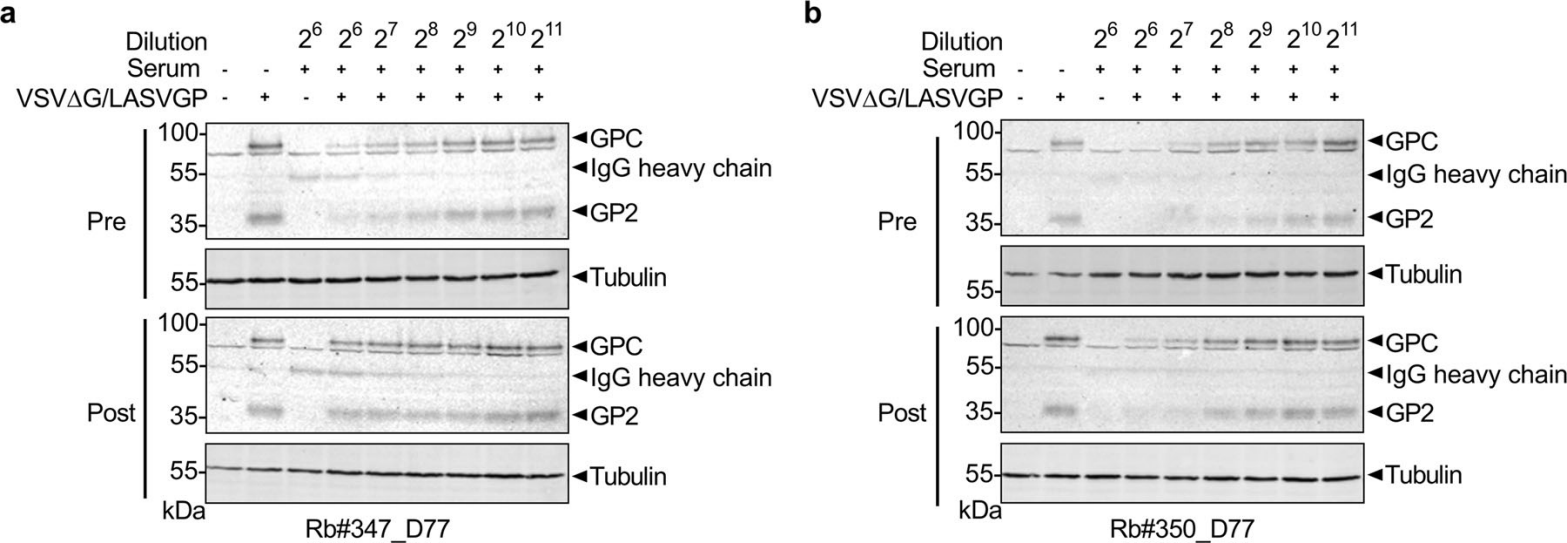
Mechanism of LASV neutralization. Pre- and postattachment inhibition assays were used to determine the mechanism of neutralization. Data are shown for D77 samples of Rb#347 (**a**) and Rb#350 (**b**). Rabbit sera were serially diluted with an initial dilution of 1:64 and incubated with VSVΔG/LASVGP either before virus attachment (pre) or after virus attachment (post) at the cell surface. Cell lysates were harvested 24 h post infection and analyzed by SDS-PAGE and western blotting. LASV GPC and GP2 were stained with rabbit α4 and an anti-rabbit Alexa Fluor 680 as a secondary antibody. Tubulin was used as a loading control.

nAbs may prevent virus infection either through blocking the attachment to host cell receptors or by interfering with postbinding events, such as internalization or viral fusion during entry. To address this, we performed pre- and postattachment neutralization assays. Antisera Rb#347_D77 and Rb#350_D77 were incubated with VSVΔG/LASVGP before or after virus binding to Vero E6 cells, and infection was measured by western blot analysis. As expected, both antibody preparations efficiently neutralized infection in a dose-dependent manner when preincubated with VSVΔG/LASVGP (Fig. 7a, b, upper panel). To determine the ability of the antibodies to neutralize infection at a postattachment step, VSVΔG/LASVGP was allowed to bind to Vero E6 cell monolayers at 4 °C for 1 h. Serially diluted polyclonal rabbit immune sera were then added to determine if they can prevent the attached virus from infecting Vero E6 cells. While we observed no neutralizing activity for antiserum Rb#347_D77, antiserum Rb#350_D77 efficiently inhibited VSVΔG/LASVGP infection in a dose-dependent manner when added after virus adsorption to the cell surface, suggesting that at least part of its neutralizing activity occurs at a postattachment step (Fig. 7a, b, lower panel). An unrelated rabbit control serum showed no inhibitory effect on VSVΔG/LASVGP infection in our pre- and postattachment neutralization assays (data not shown).

### Purification of IgG with high recovery of neutralizing activity

**Fig. 8:**
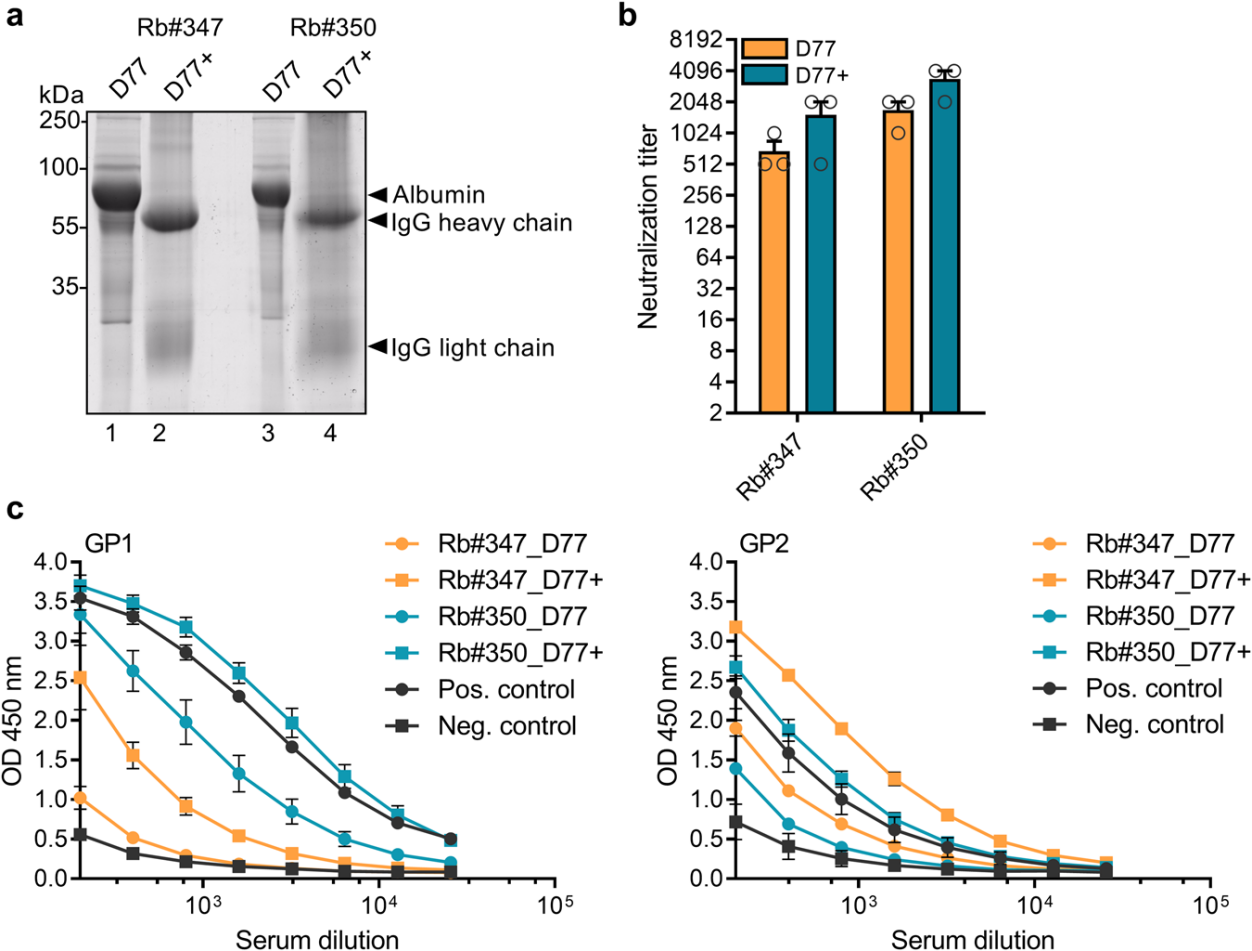
Purification and characterization of IgG preparations. **a** Using protein A affinity chromatography, IgG was isolated from rabbit sera collected 1 week after the final boost (D77). For IgG purification analysis, 3 μg of total protein from each preparation was separated by SDS-PAGE and stained with SYPRO Ruby protein gel staining. Lanes 1 and 3: initial antiserum; lanes 2 and 4: elution of protein A affinity chromatography. **b** Neutralizing antibody response against VSVΔG/LASVGP of initial antisera (D77 samples) and affinity-purified IgG (D77+ samples). Bars represent the mean, and error bars indicate the standard deviation of GMT values of three independent experiments. **c** Detection of α-LASV GP1 or GP2 antibodies using ELISA. The values shown represent the means of the reciprocal serum dilutions. Error bars are representative of the SD of OD 450 nm values (duplicate measurement of three independent experiments).

We purified rabbit IgG from the antisera using a protein A affinity column strategy and tested whether it retained functional activity. IgG purity and possible residual serum protein contaminations were assessed using SDS-PAGE followed by SYPRO Ruby protein gel staining. Figure 8a demonstrates that IgG purification yielded high levels of intact and pure IgGs for both rabbit serum samples (designated Rb#347_D77+ and Rb#350_D77+), with protein concentrations ranging from 1.23 to 7.91 mg/ml. We then assessed the ability of affinity-purified IgGs to bind to GP and to neutralize GP-mediated virus entry to shed light on changes in GP-specific IgG binding titers and measure the functional quality of the recovered IgGs. The neutralizing activity of the purified IgG preparations against VSVΔG/LASVGP was increased compared with the untreated serum samples, with geometric mean titers (GMTs) ranging from 1:1280 to 1:3072 (Fig. 8b). Consistent with the increase in nAb activity, ELISA results of purified rabbit IgG samples showed elevated binding reactivity against GP1 and GP2 over prepurified serum samples (Fig. 8c). In summary, these results demonstrate that the neutralizing properties of anti-GP IgGs were successfully recovered following protein A IgG purification.

### VLP-induced nAbs inhibit LASV GP-mediated infection of human target cells

**Fig. 9:**
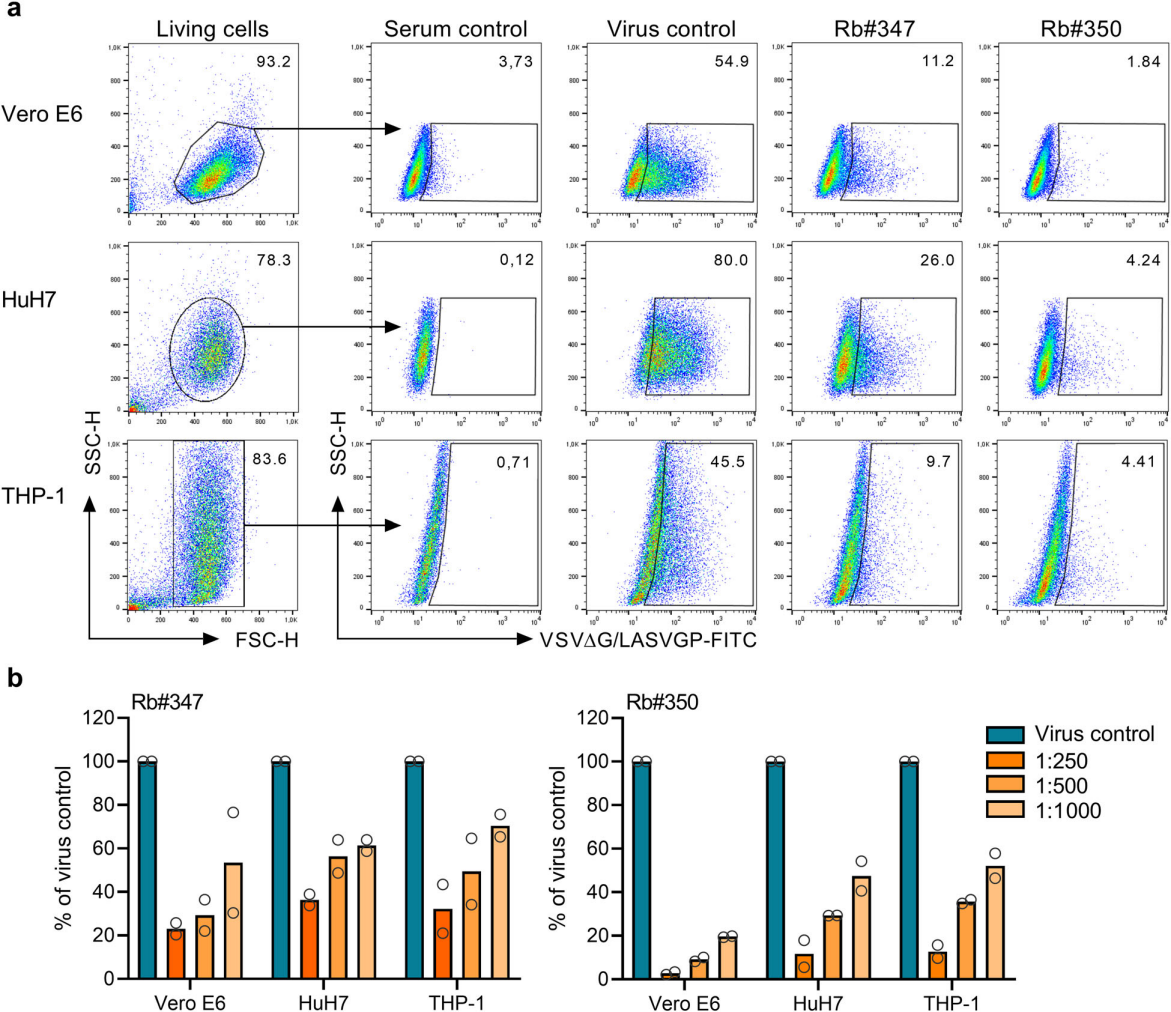
Inhibition of VSVΔG/LASVGP infection of human cells. Flow cytometry analysis was used to determine the neutralizing capacity of the IgG preparations to inhibit LASV GP-mediated infection of human cells. HuH7 cells, differentiated THP-1 cells, and, for comparison, Vero E6 cells were infected with VSVΔG/LASVGP after preincubation of the virus with serially diluted IgG preparations. Infection without sera and untreated cells served as a control. At 22 h post infection, the cells were harvested, fixed, and stained with a polyclonal guinea pig serum against VSV and an anti-guinea pig antibody labeled with FITC as a secondary antibody. **a** A representative experiment is shown to illustrate flow cytometry-based virus neutralization for Rb#347 and Rb#350 using purified IgG preparations at a dilution of 1:250. For all experiments, living cells were distinguished from cell fragments by FSC-H/SSC-H, referring to cell size and granularity in the starting population. For each cell line, untreated VSVΔG/LASVGP-infected cells were used as positive controls. Uninfected cells treated with the highest concentration of each serum were used as negative controls. These cells were then gated in the serum control that was stained only with the FITC-labeled secondary anti-guinea pig antibody to discriminate between non-infected cells and infected cells (VSVΔG/LASVGP-positive). Percentage of infected cells among the living cells is indicated. **b** Quantification of infected cells depended on the serum dilution used. Virus control was set to 100% for each cell line, and the relative number of infected cells was calculated for each serum dilution. The values shown represent the mean of two independent experiments.

LASV infects various cell types in vitro and in vivo, including monocyte-derived macrophages and dendritic cells and liver epithelial cells. The use of multiple cellular receptors likely contributes to the broad host cell tropism of LASV^10,52^. We finally aimed to evaluate the potential of the anti-LASV polyclonal antibody preparations to inhibit the infection of relevant human target cells using human hepatoma epithelial (HuH7) cells as well as differentiated THP-1 cells, a human leukemia monocytic cell line that is widely used to study monocyte/macrophage function^53^. Because the morphological characteristics of VSVΔG/LASVGP-induced cytopathic effects (CPE) varied in these cells and therefore restricted the microscopic scoring and comparison of the CPE-mediated readout, we employed a flow cytometry-based neutralization assay to circumvent these limitations. Purified IgG preparations Rb#347_D77+ and Rb#350_D77+ were twofold diluted, from 1:250 to 1:1000, and then incubated with VSVΔG/LASVGP (MOI 0.1) at 37 °C for 1 h. Then, the virus–antibody mixtures were added to the cells. At 22 h post infection, we measured the percentage of infected cells by flow cytometry analysis using a guinea pig, VSV-polyclonal antiserum as the primary antibody, and a rabbit anti-guinea pig secondary antibody labeled with fluorescein isothiocyanate (FITC). VSVΔG/LASVGP infection in the absence of neutralizing sera served as a positive control. All examined cell lines were susceptible to VSVΔG/LASVGP infection, although the infection efficiencies varied (Fig. 9a, virus control column). While the percentage of VSVΔG/LASVGP-infected Vero E6 cells reached 55%, infection of HuH7 and differentiated THP-1 cells reached 80% and 45%, respectively. Both IgG preparations efficiently reduced VSVΔG/LASVGP infectivity in all cell lines tested (Fig. 9a). When we set the virus control for each cell line to 100%, we observed a dose-dependent neutralizing effect for both IgG preparations. Similar to our observations described before, Rb#350_D77+ exhibited a greater neutralization activity against LASV GP in comparison with Rb#347_D77+ (Fig. 9b). In summary, these results demonstrate that antibodies induced by LASV GP VLP immunization are capable of neutralizing the infection of human LASV target cells.

## Discussion

LF is an acute viral disease associated with severe, life-threatening, multiple organ failure and hemorrhagic manifestations. The lack of approved medical countermeasures underscores the necessity of developing safe and effective therapeutics and vaccines. Passive immunization experiments of convalescent immunoglobulin preparations and monoclonal antibodies suggest that antibody-based therapeutics represent an effective approach for the treatment of LF. Here, we explored the potential of generating animal-derived hyperimmune sera using LASV GP expressed on the surface of VLPs as an immunogen. Our data show that the GP VLP platform is capable of inducing GP-specific antibody responses and demonstrate that this immunization approach can induce broadly nAbs.

The preservation of neutralizing epitopes is important for ensuring efficient induction of nAbs. The majority of human monoclonal antibodies that effectively neutralize LASV bind to quaternary structure epitopes, which are only expressed in the context of the properly assembled trimeric form of GP^41,42^. We employed a MDCK II cell line stably expressing full-length LASV GPC to generate VLPs that display mature GP spikes^47^. Similarities between the ultrastructural organization of the immunogenic ectodomain of GP on the surface of VLPs and authentic LASV suggest that VLP-derived GP may be capable of inducing an antibody response that is comparable with the antibody response during natural infection^49^. The robust induction of GP-binding antibodies elicited by our adjuvanted GP VLP immunization approach reported here is in line with previous work that elucidated the immunogenicity of mammalian cell-derived LASV VLPs based on co-expressed GP and matrix protein Z, or GP, Z, and NP^54^. In that study, the ability of these VLPs to induce an immune response against LASV was evaluated in mice using a prime-boost immunization schedule in the absence of an adjuvant. However, the VLPs’ protective effectiveness and capability to induce nAbs were not investigated. The binding of antibodies to both subunits GP1 and GP2 measured in our GP subunit-specific ELISA suggests that the hyperimmune sera may exhibit numerous effects. GP1 mediates binding to host cell receptors, whereas GP2 facilitates virus–host membrane fusion during entry.

Using authentic LASV and two BSL2 surrogate systems based on a replication-competent VSV expressing LASV GP and murine leukemia virus (MLV)-based LASV GP-pseudotyped particles, we measured significant neutralization activity of the hyperimmune sera. While we observed low levels of neutralization activity after a single immunization, nAb titers substantially increased after boosting. This increase suggests that multiple presentations of the antigen in its native conformation are required to produce an efficient nAb response. Immunization with adjuvanted GP VLPs induced nAb titers that were higher than nAb titers measured in LASV GP IgG-positive Guinean human serum samples. Convalescent blood therapy has been suggested to improve the survival rate of LF patients. However, high nAb titers and antigenic matching were required for protection^21,34^. It will be interesting to determine whether GP VLPs can induce high titers of nAbs that are sufficient to give protection against experimental LASV challenges in animal models. Affinity chromatography methods using immobilized GP as a ligand may be used to improve the neutralizing activity of hyperimmune sera-derived antibodies.

The high degree of sequence divergence of LASV genomes is a major challenge for the development of an effective pan-LASV vaccine and immunotherapeutic intervention strategies. An ideal vaccine or antibody-based therapeutic will need to confer protection against all circulating LASV strains. Currently, LASV strains are grouped into four established lineages. Lineages I, II, and III circulate in Nigeria, whereas lineage IV circulates in several other West African countries including Guinea, Sierra Leone, and Liberia^55^. In addition, several putative lineages have been proposed for strains circulating in Mali and Côte d’Ivoire^56^ (lineage V), Nigeria^57^ (lineage VI), and Togo^58^ (lineage VII). Despite this genetic diversity, some human monoclonal antibodies with broadly cross-neutralizing activity have been described^41^. Using neutralization assays with authentic LASV, the hyperimmune sera from VLP-immunized animals showed potent neutralizing activities against the homologous LASV strain Josiah (lineage IV), and were also capable of cross-neutralizing the infection of a clinical isolate from lineage II that was recently isolated from an LASV patient during the 2018 LF outbreak in Nigeria^50^. As determined by a pseudotype-based neutralization assay, it is encouraging that our VLP immunization approach using GP from the prototypic strain Josiah elicited broadly reactive antibodies capable of inhibiting virus entry of five different LASV lineages (I–V). Our results suggest that the VLP immunization elicited antibodies that bind to conserved epitopes associated with cross-neutralization of multiple LASV lineages.

Antibodies can block viral infection at various steps during virus entry. Both rabbit immune sera inhibited virus infection when preincubated with VSVΔG/LASVGP, suggesting that the serum antibodies can block the interaction between GP and host cell surface receptors. In addition, antibodies may also be capable of blocking infection at a postattachment step. We found that the antiserum Rb#350 also inhibited VSVΔG/LASVGP infection when added after virus adsorption to the cell surface, suggesting that part of its neutralizing activity occurs at a postattachment step. These antibodies may inhibit virus internalization or block the conformational changes of GP required to mediate fusion with the host membrane. For example, structural analyses revealed that the human monoclonal nAb 37.7H neutralizes LASV by stabilizing GP in the prefusion conformation, thereby inhibiting the conformational changes required for intracellular LAMP1 binding and subsequent virus fusion^42^. Structure-based epitope mapping of polyclonal antibodies elicited by LASV GP VLP immunization may help to elucidate the mechanisms underlying GP binding and neutralization^59^. Furthermore, using HuH7 cells and differentiated THP-1 cells as representative cell culture models, we found that the rabbit immune sera were able to inhibit in vitro virus infection of human epithelial liver cells and macrophages. Particularly, immune cells are the early target cells of human LASV infection^60^. Therefore, our VLP-elicited antibodies may have the potential to block virus dissemination in vivo by inhibiting virus replication at the early stage of the disease.

We show an extensive nAb response here despite the use of fully glycosylated GP as an immunogen. These results were somewhat unexpected because previous studies have suggested that the native GP is a poor immunogen for efficiently inducing nAbs^18,26^. This poor outcome has been attributed to the presence of an extensive glycan shield^19^. Studies in mice have shown that changes in the number of N-glycans alter viral neutralization sensitivity from resistant to potently neutralizing^18^. Although the removal of individual N-glycans improved the immunogenicity and induction of nAbs, the contribution of N-glycans to GP folding and maturation makes them critical components for the induction of a biologically relevant GP-specific antibody response^15^, suggesting that the native glycosylation profile may be needed for the effective induction of nAbs.

Passive antibody therapy has long been used in the treatment and postexposure prophylaxis of various diseases, including viral infections and the development of heterologous polyclonal antibody preparations obtained from hyperimmunized animals against several highly pathogenic viruses such as Ebola, Junín, and *Nipah*^40,46,61,62^. Our VLP-based approach combines several advantages for therapeutic antibody production. VLPs morphologically resemble authentic virions that display antigens in their native conformation in a highly ordered and repetitive manner capable of inducing strong immune responses^63^. Most human antibodies that neutralize LASV bind to quaternary structure epitopes containing regions from both GP1 and GP2. LASV VLPs display a correctly folded and conformationally relevant GP, which means that they have advantages over GP-subunit vaccines that lack higher-order protein structures and epitopes displayed on the intact virus^64,65^. As hyperimmunized serum contains a mixed population of antibodies, polyclonal antibody preparations can bind and neutralize multiple epitopes on GP, which may also reduce the emergence of viral escape mutants. VLPs do not contain a viral genome, so they are not replication competent and therefore not infectious. Therefore, VLPs do not need to be inactivated during the manufacturing process, which may compromise vaccine efficacy due to potential effects on protein structure and conformational epitopes. Furthermore, in the case of newly emerging, phylogenetically, and antigenically distinct LASV, cell culture-based GP VLPs can be rapidly generated based on available sequence information as antigens and animal-derived polyclonal antisera can be produced in scalable quantities. Thus, hyperimmune sera or hyperimmune immunoglobulin preparations are an attractive alternative to human monoclonal antibodies isolated from LF survivors in the early phase of an outbreak when convalescent donor material may not be readily available. Rabbit-derived immunoglobulin preparations are used for the treatment of certain conditions and are, in general, well tolerated. For example, rabbit-derived antithymocyte globulin is licensed for clinical use for the prevention and treatment of acute rejection in organ transplantation^66^.

Several vaccines under development against emerging viruses are based on the VLP platform^67^, providing a rationale for the use of LASV GP-derived VLPs as a vaccine candidate. An effective vaccine against LASV is likely to require the induction of robust antibody and T-cell responses. We demonstrate that GP VLPs induced robust nAb responses in rabbits, yet their capacity to induce efficient cellular responses and cell-mediated protection against LASV remains to be determined. In general, the good safety profile makes VLP-based vaccines an appropriate choice for children, immunocompromised individuals, and pregnant women. The WHO has identified pregnant women as a primary target population for LASV vaccination, because LF is particularly severe in pregnancy, with high rates of maternal and fetal death^68^.

Our study has several limitations. We used the Sigma Adjuvant System as an immunostimulant, but we did not investigate the immunogenicity of GP VLPs and the induction of nAbs in the absence of an adjuvant. Nor did we assess the capacity of other adjuvants to enhance the antibody response. Alternative adjuvant formulations may have the potential to induce a more vigorous production of LASV-nAbs. Future studies are needed to explore the effectiveness of different adjuvant systems systematically^69,70^. Such data will guide the selection of an optimal adjuvant formulation for GP VLP-based immunization strategies. Passive transfer of human monoclonal neutralizing anti-GP antibodies confers protection against LF in animal models^37,71^. While our results indicate that the GP-derived VLPs described in this study are potent at inducing nAbs against LASV, future studies need to evaluate their therapeutic efficacy in animal models. Furthermore, we have used canine cells to generate LASV GP VLPs to ensure structurally authentic mammalian glycosylation. Our enzymatic deglycosylation assays revealed a glycosylation pattern of VLP-derived GP similar to that of naturally occurring LASV virions released from human and rodent cells. While the N-linked glycan biosynthesis processing pathway is highly conserved across mammalian cell types, species-specific variations exist that may influence the immunogenicity of the target antigen. Current research efforts address the use of human cell lines for GP VLP manufacture because only human cell lines can produce proteins that contain posttranslational modifications (PTMs) similar to those seen in humans^72^. Using human cell lines would offer the advantage of producing a human-specific glycosylation pattern of PTMs, as compared with those produced in animal cell lines.

In conclusion, our results demonstrated that LASV GP-induced VLPs in combination with a squalene-based adjuvant formulation were immunoreactive in an animal model and capable of inducing potent broadly nAb responses. These data warrant further research of heterologous animal-derived hyperimmune sera as a potential treatment option against highly pathogenic LASV.

## Methods

### Cells, plasmids, and viruses

African green monkey kidney cells (Vero E6, ATCC CRL-1586) and human hepatoma cells (HuH7, ATCC CCL-185) were maintained in Dulbecco’s modified Eagle’s medium (DMEM, Sigma-Aldrich). MDCK II cells (ATCC CCL-34) were maintained in modified Eagle’s medium (MEM, Sigma-Aldrich). Each medium was supplemented with 10% (v/v) fetal bovine serum (FBS, Invitrogen), 2 mM l-glutamine (Sigma-Aldrich), and 50 U/ml penicillin and 50 µg/ml streptomycin (Sigma-Aldrich). Human embryonic kidney 293T (HEK293T, ECACC 12022001) cells were grown in Dulbecco’s modified essential medium (Invitrogen) supplemented with 10% FBS and 0.1 mM nonessential amino acids (NEAA, Gibco). Baby hamster kidney cells (CVCL_1915) were maintained in Glasgow’s MEM (Gibco) supplemented with 10% (v/v) newborn calf serum (Gibco), 2 mM l-glutamine (Sigma-Aldrich), 50 U/ml penicillin and 50 µg/ml streptomycin (Sigma-Aldrich), 1 × NEAA (Gibco), and 5% tryptose phosphate broth (Gibco). The THP-1 cells (ATCC-TIB-202) were maintained in RPMI 1640 medium (PanBiotech) supplemented with 10% FBS and 2 mM l-glutamine, 50 U/ml penicillin and 50 µg/ml streptomycin, and 1 × NEAA and sodium pyruvate (Gibco). THP-1 cells (4 × 10^5^/well) were differentiated using 200 nM phorbol 12-myristate 13-acetate (PMA, Sigma-Aldrich)^73^. PMA was removed after 2 days of treatment. Cells were then grown for an additional 5 days in supplemented RPMI 1640. All cell cultures were grown under standard conditions in a humidified 37 °C, 5% CO_2_ incubator.

For vectorial expression of arenavirus GPs in eukaryotic cells, we used the mammalian expression vector pCAGGS^74^. The pCAGGS plasmid encoding the ORF of full-length GPC of LASV strain Josiah (GenBank NC_004296, lineage IV) and LCMV strain WE (GenBank AAA46265), respectively, were described earlier^47,75^. The ORF of LASV Togo GPC (GenBank LT601602, lineage VII^76^) was amplified from viral RNA using reverse transcription (RT)-PCR and cloned into pCAGGS. The GPC ORFs of LASV strain Pinneo (GenBank KM822128, lineage I), LASV strain 803213 (GenBank AF181854, lineage II), and LASV strain AV (GenBank AF246121, lineage V) were commercially synthesized (BaseClear B.V., The Netherlands) and cloned into pCAGGS. The sequences of the GP ORFs were confirmed by DNA sequencing. Vero E6 cells were transfected using TransIT-LT1 (Mirus) according to the manufacturer’s protocol.

For LASV stock production, the LASV strain Josiah (GenBank AY628203) and the LASV human clinical isolate LASV/NGA/2018/IRR/015 (GenBank MK117963)^50^ were grown on Vero cells. Viral titers were determined by an immunofocus assay, or by defining the 50% tissue culture infectious dose (TCID_50_).

For immunofocus assay, Vero cells in 24-well plates were inoculated with 200 µl of serial tenfold dilutions of LASV. After 1 h, the inoculum was removed and 1%-methylcellulose medium overlay was added. The cells were incubated for 5 days and then fixed with 4% formaldehyde, permeabilized with 0.5% Triton X-100, and blocked with 10% FCS. Cell foci were detected with LASV NP-specific mouse monoclonal antibody 2F1 and a peroxidase-labeled anti-mouse IgG as secondary antibody. Foci were visualized with tetramethylbenzidine and counted^77^. For TCID_50_ assay, Vero cells were grown in 96-well plates to 60% confluence. Cells were inoculated with tenfold serial dilutions of supernatant from LASV-infected Vero cells. The assay was evaluated at 7–9 days post infection. TCID_50_ values were calculated using the Reed–Muench method^78^. All experiments with infectious LASV were performed in the BSL4 facilities at the Institute of Virology of the Philipps-University Marburg, Marburg, and the Bernhard-Nocht Institute for Tropical Medicine, Hamburg, in compliance with German regulations.

The recombinant VSV expressing the GPC derived from LASV strain Josiah in place of the original VSV G (VSVΔG/LASVGP) was grown in Vero E6 cells^79^. The viral titer of VSVΔG/LASVGP was determined by plaque assay using an Avicel RC-591 overlay. The day before the assay, 3.3 × 10^5^ Vero E6 cells/well were seeded and cultivated until reaching a confluence of 100%. Tenfold serial virus dilutions in DMEM without FBS were performed and 400 µl/well were added to the cells in 12-well tissue culture plates. For the overlay, 2.4% Avicel in phosphate-buffered saline (PBS) buffer solution was mixed with an equal volume of MEM with 4% FBS and 4 mM l-glutamine, 100 U/ml penicillin, and 100 µg/ml streptomycin. After incubation for 1 h at RT with gentle agitation, 800 µl/well of 1.2% Avicel overlay were added, and the cells were incubated at 37 °C for 48 h. To fix the cells, the Avicel overlay was aspirated and 10% formaldehyde solution containing 0.1% (w/v) crystal violet was added and incubated for 30 min at RT. Viral titers were expressed as plaque-forming units (PFU).

For the generation of pseudotype viruses, five GPC constructs (all in the pCAGGS expression plasmid) representing five different LASV lineages were used. The LASV GPCs used were: lineage I, LASV/LP/NIG/69/H, GenBank AF181853; lineage II, LASV/803213/NIG/74/H, GenBank AF181854; lineage III, LASV/GA391/NIG/74/H, GenBank X52400; lineage IV, Josiah, GenBank NP_694870; and lineage V, LASV/AV/IC, GenBank AF246121. The MLV Gag-Pol packaging vector (phCMV-5349) and luciferase encoding reporter plasmid (pTG126) have been reported previously^80^. All plasmids were grown and purified using endotoxin-free Genelute^TM^ HP Midiprep kits (Sigma). DNA quality was assessed by agarose gel electrophoresis and quantified by spectrophotometry at 260/280 nm using a Nanodrop (Thermo). LASVpv were generated based on previously described protocols^80^. Briefly, 1.5 × 10^6^ HEK293T cells were seeded overnight in a 10 cm diameter Primaria-coated dish (Corning). Transfections were performed with 2 µg each of the MLV Gag-Pol packaging vector, luciferase reporter plasmid, and plasmid-encoding LASV GPC using 24 µl cationic polymer transfection reagent (polyethylenimine), in the presence of Optimem (Gibco), and the media replaced with 10 ml complete DMEM after 6 h. A no-envelope control (empty pseudovirus) was used as a negative control in all experiments. Supernatants containing LASVpv were harvested at 72 h post transfection and filtered through 0.45-μm-pore-size membranes. The generated LASVpv can achieve a single-round infection in HuH7 target cells and contains a luciferase reporter gene that can be expressed in infected cells.

### Antibodies

LASV GP subunits were detected using the monoclonal mouse antibody, designated AC1, specific for the GP1 subunit (kindly provided by M.C. Georges-Courbot, Unit of Biology of Viral Emerging Infections, Institute Pasteur, Lyon, France), a monoclonal human antibody designated 37.7H (absolute antibody) or polyclonal antibodies raised in rabbits: α231 recognizing the C-terminal GP1 domain, α3 recognizing the N-terminal GP2 ectodomain, and α4 detecting the cytoplasmic domain of GP2^81,82^. A mouse monoclonal anti-LASV nucleoprotein NP antibody, designated 2F1, was used for the detection of infected foci by immune focus assays^77^. Guinea pig VSV-polyclonal antiserum was kindly provided by W. Garten (Institute of Virology, Marburg, Germany). Mouse monoclonal tubulin antibody was obtained from SIGMA Life Science. For western blot analysis, an anti-rabbit secondary antibody labeled with Alexa Fluor 680 (Invitrogen) and an anti-mouse secondary antibody labeled with Alexa Fluor Plus 800 (Invitrogen) was used. Anti-rabbit secondary antibodies labeled with Alexa Fluor 568 or 488 (both from Invitrogen) were used for immunofluorescence staining and a secondary antibody labeled with horseradish peroxidase (HRP, DAKO) was used for IPMA and indirect ELISA. An anti-human secondary antibody labeled with FITC (DAKO) was used for immunofluorescence staining. An anti-guinea pig secondary antibody labeled with FITC (DAKO) was used for flow cytometry staining. An anti-mouse secondary antibody labeled with HRP (Jackson Immuno Research Laboratories) was used for immune focus assays.

### Human serum samples

Guinean human sera were collected in a highly LASV-endemic area of Guinea in 2014 by venipuncture from healthy individuals (≥18 years) after written informed consent was obtained (Fichet-Calvet and Magassouba, unpublished data). Approval for the investigation was obtained from the National Ethics Commission of Guinea (permit no. 12/CNERS/12). A total of 96 European human serum samples from healthy anonymous donors were used to confirm specificity of the LASV GP IgG ELISA.

### Production of VLPs

The generation of MDCK II cell lines stably expressing LASV GP has been described previously^47^. For production of GP-derived VLPs, MDCK II LASV GP stable cell lines were seeded in 175 cm^2^ tissue culture flasks and cultured at 37 °C for 4 days. After reaching a confluence of 100%, the medium were replaced by MEM supplemented with 2% FBS, 2 mM l-glutamine, and 50 U/ml penicillin and 50 µg/ml streptomycin, and the cells were incubated for an additional 4 days. Culture supernatants were harvested and clarified twice by centrifugation at 3345 × *g* for 10 min at 4 °C. VLPs were then pelleted by ultracentrifugation through a 20% (w/v) sucrose cushion for 1.5 h at 110,000 × *g* and 4 °C. The pellets containing VLPs were resuspended in sterile PBS and stored at 4 °C. The VLPs were freshly prepared prior to each immunization. VLP preparations used for western blot analysis or electron microscopy (EM) were stored at −80 °C until further analysis. The total protein concentration of the VLP preparations was 2.2 ± 0.2 mg/ml, as determined by a Pierce BCA Protein Assay kit with a bovine serum albumin (BSA) standard (Thermo Scientific).

### Treatment with endoglycosidases

MDCK II LASV GP cells, or MDCK II cells, HuH7 cells, and BHK cells infected with LASV Josiah at a multiplicity of infection (MOI) of 0.1 were incubated for 5 days. Cell culture supernatants were clarified by low centrifugation at 3345 × *g* for 10 min at 4 °C. VLPs and virus particles were then harvested by ultracentrifugation through a 20% (w/v) sucrose cushion for 1.5 h at 110,000 × *g* and 4 °C. The pellets containing GP VLPs or LASV virions were resuspended in sterile PBS, followed by the addition of a reducing SDS sample buffer. Samples were treated with either EndoH (New England Biolabs) or N-glycosidase F (PNGaseF, New England Biolabs) according to the instructions of the manufacturer or left untreated. Samples were analyzed by SDS-PAGE and western blot analysis.

### SDS-PAGE and western blot analysis

For verification of sample quality and purity, 0.4 µg recombinant LASV GP1 and GP2 proteins and IgG purified from rabbit blood (3 µg of total protein) were separated by SDS-PAGE using 12% polyacrylamide gels and stained with SYPRO Ruby protein gel staining (Invitrogen) according to the manufacturer’s instructions. Samples were analyzed using the ChemiDoc Imaging System (Bio-Rad). The cell lysates of MDCK II cells and MDCK II cells stably expressing LASV GPC, as well as purified VLP and authentic LASV virion preparations, were separated by SDS-PAGE using 12% polyacrylamide gels and transferred to nitrocellulose blotting membranes (GE Healthcare Life Science) for western blot analysis. The Odyssey Infrared Imaging System (LI-COR Biosciences) was used for visualization and quantification of detected proteins. All gels/blots were derived from the same experiment and were processed in parallel. In case several gels were used to fit all samples, they all derive from the same experiment and were processed in parallel. Uncropped images of the original gels/blots are presented in the Supplementary Information.

### Immunofluorescence assays

A total of 6 × 10^4^ Vero E6 cells/well were seeded on coverslips and transfected with 1 µg pCAGGS expression plasmids encoding various Old World arenavirus GPs, and 4.7 × 10^4^ MDCK II cells stably expressing LASV GP were seeded on coverslips and cultivated until reaching a confluence of 80%. Expression of GP was analyzed 24 h post transfection in Vero E6 cells and 24 h post seeding in MDCK II LASV GP cells. For the staining of fixed antigens, the medium were aspirated and cells were incubated with 4% PFA for 20 min at RT and then incubated with 0.1 M glycine for 10 min at RT. Before primary antibody incubation, the cells were permeabilized with 0.1% Triton X-100 for 10 min, and nonspecific sites of antibody adsorption were blocked with 0.2% BSA (Aurion) solution for 20 min at RT. For the staining of unfixed antigens, cell culture medium were aspirated, and the cells were incubated with the primary antibody. Then, the cells were fixed with 4% PFA for 20 min at RT, incubated with 0.1 M glycine for 10 min, and finally permeabilized with 0.1% Triton X-100 for 10 min at RT. LASV GP was stained with primary rabbit antibodies diluted 1:100 or 1:200 in PBS and incubated for 1 h at RT. An anti-rabbit antibody conjugated with Alexa Fluor 488 or 568 was used as a secondary antibody. Cell nuclei were stained with 0.5 µg/ml 4′,6-diamidino-2-phenylindole (DAPI, Invitrogen). Finally, cells were washed three times with PBS and subsequently mounted in Fluoroshield (Sigma-Aldrich) for imaging. Images were taken on a Leica confocal microscope or with Zeiss Axiovert 200M with ×63 objectives. Image processing was performed using Fiji ImageJ software.

### Electron microscopy

Transmission EM was performed with purified GP VLPs after fixation with 1% PFA for 1 h. VLP suspension was applied to formvar-coated nickel grids and incubated for 10 min. After brief washing in PBS, the samples were negatively stained with 2% phosphotungstic acid. For on-section immuno-EM, stable transfected and nontransfected MDCK II cells were slowly grown in a six-well tissue culture plate until reaching a confluence of 100% and fixed at a final concentration of 0.1% glutaraldehyde and 2% formaldehyde by addition of double-strength fixative to the growth medium. The cells were pelleted, treated with 0.25% tannic acid, and further fixed with 1% osmium tetroxide containing 1.5% ferricyanide. Pellets were dehydrated in a graded ethanol series and infiltrated with LR White containing 2% benzoyl peroxide at 4 °C, followed by polymerization at 53 °C for 24 h in gelatin capsules. Ultrathin sections of 70 nm were collected on formvar/carbon-coated nickel grids. For immunogold labeling, the sections were blocked in 1% BSA in PBS and then incubated for 1 h with rabbit sera Rb#347_D77 and Rb#350_D77, respectively, diluted 1:20 in PBS containing 1% BSA. After washing with PBS, the grids were incubated for 1 h in protein A conjugated to colloidal gold (PAG 6 nm, Aurion), washed with PBS, treated with 0.25% glutaraldehyde, washed with water, and finally air-dried. All electron micrographs were acquired using a JEOL JEM1400 with a TVIPS TemCam F416 camera.

### Immunization

All animal experiments were carried out in compliance with the regulations of German animal protection laws and authorized by the Regierungspräsidium Darmstadt, Germany. New Zealand White rabbits (*Oryctolagus cuniculus*) were purchased from Charles River Germany. For generation of rabbit LASV antisera, animals were immunized intramuscularly with 300 µg of LASV GP VLPs mixed in a 1:1 ratio (v/v) with Sigma Adjuvant System, and boosted 4, 7, and 10 weeks later. Blood samples were collected before each boost from the marginal ear vein on days 0, 28, 49, and 70, and animals were sacrificed 77 days post immunization by exsanguination.

### Antisera purification

Total IgG was purified from rabbit antisera using the Antibody Serum Purification Kit (Protein A) from Abcam according to the manufacturer’s instructions. The antibody concentration was determined using a Pierce BCA Protein Assay kit with a BSA standard (Thermo Scientific) and measured in a NanoPhotometer NP80 (Implen). The purity of IgG preparations was assessed by 12% SDS-PAGE and SYPRO Ruby protein gel staining (Invitrogen).

### Quantification of total IgG antibodies by IPMA

For determination of LASV GP-specific antibodies by IPMA, 2 × 10^2^ Vero E6 cells/well were seeded in 96-well tissue culture plates and infected with 800 PFU/well of VSVΔG/LASVGP, or with the same amount of DMEM as a control. Cells were incubated for 24 h at 37 °C before fixation with 4% PFA and permeabilized with 0.1% Triton X-100. Serial twofold dilutions of an initial 1:16 dilution of immunized rabbit antisera or LASV GP-specific polyclonal antibodies α4 as a positive control were added to the fixed cells and incubated for 1 h at RT, followed by incubation with an HRP-coupled secondary antibody, and visualized by staining with TrueBlue (KPL). Total antibody titers were calculated as a mean of the reciprocal value of the last serum dilution at which positive staining was detected from three independent experiments.

### Quantification of total IgG antibodies by ELISA

Total IgG antibody responses against LASV GP in human serum samples were determined by ELISA. Briefly, high binding ELISA microtiter plates (Greiner Bio-One) were coated with 2.5 µg/ml of purified LASV GP VLPs and incubated for 20 h. Following washing in PBS/0.1% Tween20 (PBST) and blocking (PBS/5% milk powder), 1:200 dilutions of serum sample were added to the plates and incubated for 1 h. Polyclonal-HRP antibody (1:1000, DAKO) in conjunction with TMB substrate (KPL) was used to develop the reaction. OD was determined at 450–650 nm (Synergy H1 Multi-Mode Microplate Reader, BioTek). Each sample was analyzed in duplicate and the mean OD value of the mock preparation was subtracted. A standard curve generated from human monoclonal antibody 37.7H was included with each measurement. The cut-off was calculated as the mean plus twofold standard deviation of the value of 96 negative human plasma samples from healthy German blood donors, and the total antibody titers were expressed as OD 450 nm.

Total IgG antibody responses against LASV GP in rabbit immune sera were determined by ELISA using recombinant GP1 and GP2 of LASV strain GA391 (The Native Antigen Company) with human Fc-tag were for coating. The recombinant proteins were diluted in sterile PBS, and ELISA microtiter plates were coated with a 2.5 µg/ml protein solution. Antisera from immunized rabbits, or anti-GP α231 and α3 antibodies as positive controls, were added at the respective dilution and detected by a polyclonal anti-rabbit HRP-coupled secondary antibody (DAKO). After staining with TMB (KPL) for 10 min and addition of the stop solution (KPL), the OD was measured at 450 nm (Epoch Microplate Spectrophotometer, BioTek). Each sample was analyzed in duplicate, and the mean OD values of three independent measurements were determined. The cut-off was calculated as the mean plus 10% of the value of naïve rabbit sera, and the total antibody titers were expressed as OD 450 nm. Statistical analysis was performed using GraphPad Prism software version 8. Statistical significance was calculated using an unpaired *t*-test (*p value < 0.05).

### Neutralization assay using authentic LASV

Immunofocus reduction neutralization assays were carried out to determine the functional activity of antibodies against authentic LASV (LASV Josiah, lineage IV or LASV/NGA/2018/IRR/015, lineage II) under biosafety level 4 containment conditions. Therefore, Vero 76 cells were seeded in 96-well plates at a density of 12,000 cells/well 1 day before the neutralization assays. Rabbit antisera were complement inactivated for 30 min at 56 °C and diluted in a twofold dilution series with a starting dilution of 2^2^. Diluted sera, or monoclonal antibody 37.7H as positive control (initial concentration of 125 µg/ml), were mixed with 1200 FFU virus and incubated for 60 min at 37 °C. Cell culture supernatant was removed, and 50 µl of the antibody–virus mixtures were added for 60 min at 37 °C to the cells. After this, the inoculum was removed and 200 µl of 1% methylcellulose overlay medium were added. At 24 h post infection, the overlay medium were removed, and the cells were fixed in 4% formaldehyde in PBS for 30 min. After permeabilization with 0.5% Triton-X-100 for 20 min and blocking with 2% FCS in PBS for 30 min, the infected cells were visualized through staining with a mouse anti-LASV NP antibody, followed by incubation with an anti-mouse HRP-labeled secondary antibody and TMB. Spots were counted using an ELISpot reader (AID ELISpot Reader Version 7.0). IC_50_ values were calculated with a sigmoidal 4PL regression model using GraphPad Prism 8 software.

### Neutralization assay using VSVΔG/LASVGP

Replication-competent VSVΔG/LASVGP was used as a surrogate model system to study LASV neutralization under biosafety level 2 containment conditions. Complement-inactivated sera, or monoclonal antibody 37.7H as positive control (initial concentration of 62.5 µg/ml), were serially diluted, from 2^3^ to 2^15^, in 96-well tissue culture plates. Then, 25 PFU of VSVΔG/LASVGP were added to the serum dilutions, and after incubation for 1 h at 37 °C, the virus–antibody mixtures were added to confluent Vero E6 cells. The cytopathic effect (CPE) was evaluated 2 days after infection. Neutralization is defined as the reduction of CPE in serum dilutions compared with CPE in virus controls. Neutralization titers were calculated as GMTs of four replicates (reciprocal value).

### Pseudotype-based virus neutralization assay

For infectivity and neutralization testing of LASV pseudotypes, 1.5 × 10^4^ HuH7 cells/well were plated in white 96-well tissue culture plates and incubated overnight at 37 °C. The following day, LASV pseudotypes were mixed with appropriate amounts of heat-inactivated sera or monoclonal antibody 37.7H as positive control (initial concentration of 50 µg/ml) and then incubated for 1 h at RT before being added to HuH7 cells for 4 h. Following this, sera and media were discarded, and 200 µl DMEM were added to the cells. After 72 h, the medium were discarded, and cells were lysed with cell lysis buffer (Promega) and placed on a rocker for 15 min. Luciferase activity was then measured in relative light units using a FLUOstar Omega plate reader (BMG Labtech) with MARS software. Each sample was tested in triplicate. Neutralizing activities were reported as the serum dilution level corresponding to 50% inhibitory dilution (ID_50_) values and were calculated by nonlinear regression (GraphPad Prism version 8), using lower and upper bounds (0 and 100% inhibition) as constraints to assist curve fitting.

### Flow cytometry-based virus neutralization assay

For the flow cytometry-based neutralization test, 4 × 10^5^ cells/well of Vero E6, HuH7, and differentiated THP-1 cells were seeded in six-well tissue culture plates. Purified IgG preparations (Rb#347_D77+ and Rb#350_D77+) were serially diluted from 1:250 to 1:1000, and incubated for 1 h at 37 °C with an equal volume of VSVΔG/LASVGP using an MOI of 0.1. The virus–antibody mixture was added to the cell monolayers for 22 h. Untreated VSVΔG/LASVGP-infected cells were used as virus controls. Cells were gently harvested by trypsinization, fixed with 4% PFA for 20 min at RT, and permeabilized with FACS buffer (PBS, 3% FBS, 2 mM EDTA, 0.001% sodium azide) supplemented with 0.5% saponin for 30 min at RT. Samples were incubated with guinea pig VSV-polyclonal antiserum at a dilution of 1:1000 and then with an anti-guinea pig secondary antibody labeled with FITC at a dilution of 1:1000 for 1 h at 4 °C each. After each incubation step, cells were washed twice with FACS buffer and finally subjected to flow cytometry analysis. Cells were analyzed with BD FACSCalibur, and data sets were evaluated using the FlowJo v10.0 software package.

### Pre- and postattachment assay

Functional characterization of neutralizing activity was determined by virus neutralization assay using VSVΔG/LASVGP and subsequent western blot analysis. For preattachment neutralization, the rabbit immune sera were serially diluted from 2^6^ to 2^11^, and 300 PFU units of the virus were added to each serum dilution. Following incubation at 4 °C for 1 h, the mixture was added to Vero E6 cells in a 12-well tissue culture plate and incubated for 1 h at 4 °C. After a further incubation at 37 °C for 1 h, the inoculum was removed and replaced by DMEM with 2% FBS. For postattachment neutralization, 300 PFU units of VSVΔG/LASVGP were added to Vero E6 cells in a 12-well tissue culture plate and incubated for 1 h at 4 °C. The serially diluted rabbit immune antisera were then added to the attached viruses. After incubation at 4 °C for 1 h, and additional incubation at 37 °C for 0.5 h, the inoculum was removed and, after washing the cells with PBS, fresh DMEM supplemented with 2% FBS was added. Cells were then incubated at 37 °C with 5% CO_2_, and the cell lysates were harvested 24 h post infection. For the detection of viral proteins, the samples were analyzed by SDS-PAGE and western blot analysis. The Odyssey Infrared Imaging System (LI-COR Biosciences) was used for visualization of detected proteins.

### Reporting summary

Further information on experimental design is available in the Nature Research Reporting Summary linked to this article.

## Supplementary information

Supplementary Information

Reporting Summary

## Data Availability

The data reported in this paper are available from the corresponding author upon request.
