# Supplementary material for: Adjuvant formulated virus-like particles expressing native-like forms of the Lassa virus envelope surface glycoprotein are immunogenic and induce antibodies with broadly neutralizing activity
Source: NPJ Vaccines. 2020 Aug 4;5:71. doi: 10.1038/s41541-020-00219-x (PMC7403343; doi:10.1038/s41541-020-00219-x)
Supplement: Supplementary file 1 — Supplementary Information [file 41541_2020_219_MOESM1_ESM.pdf]

**Supplementary Information**

# Adjuvant formulated virus-like particles expressing native-like forms of the Lassa virus envelope surface glycoprotein are immunogenic and induce antibodies with broadly neutralizing activity

Helena Müller, Sarah Katharina Fehling, Jens Dorna, Richard A. Urbanowicz, Lisa Oestereich, Yvonne Krebs, Larissa Kolesnikova, Martin Schauflinger, Verena Krähling, N'Faly Magassouba, Elisabeth Fichet-Calvet, Jonathan K. Ball, Andreas Kaufmann, Stefan Bauer, Stephan Becker, Veronika von Messling, Thomas Strecker

Supplementary Figures S1-4: Full-length pictures of gels/blots presented in the main figures.

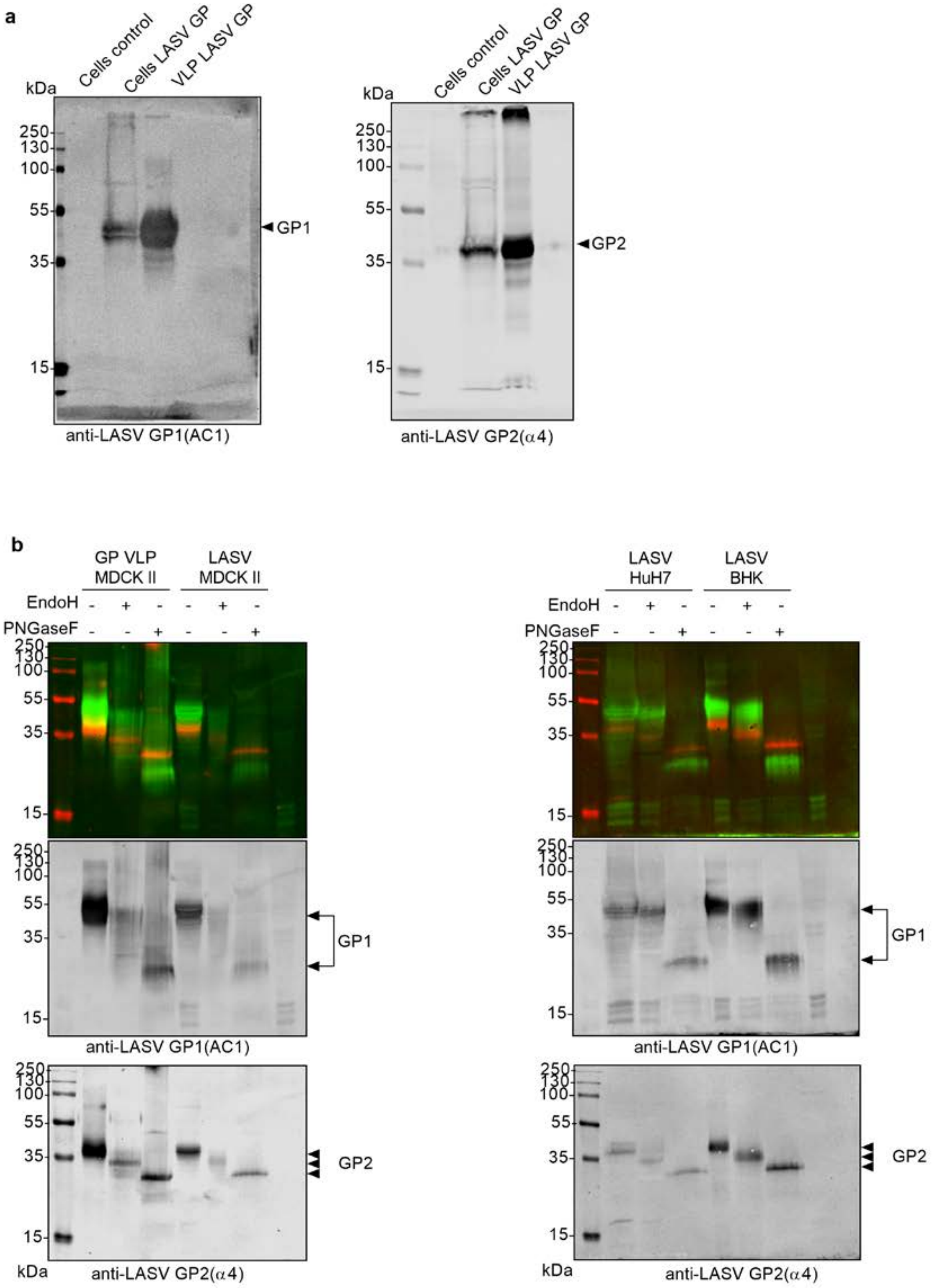


**Supplementary Fig. S1:** (a) Full-length pictures of the blots presented in Figure 1b. (b) Full-length picture of the blots presented in Figure 1d.

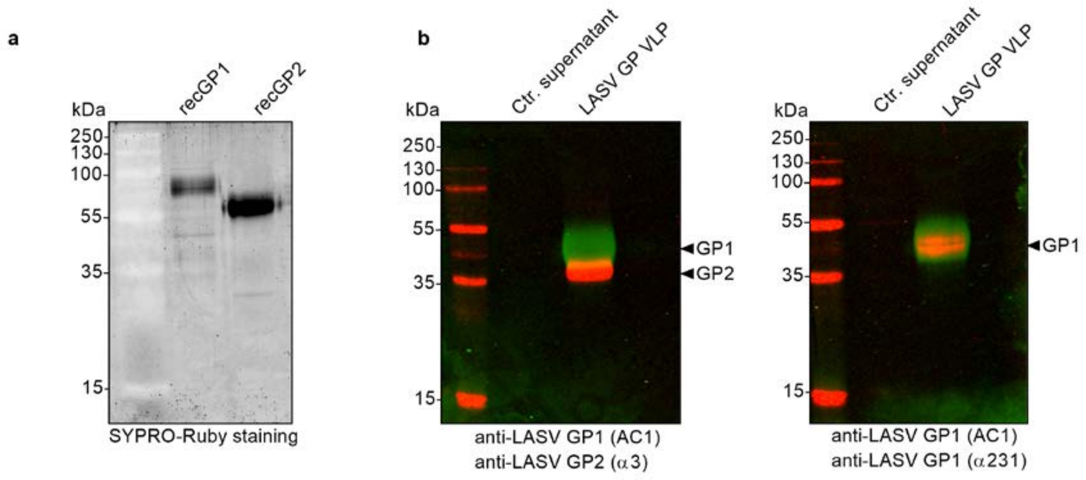


**Supplementary Fig. S2:** (a) Full-length picture of the gel presented in Figure 3b. (b) Full-length pictures of the blots presented in Figure 3c.

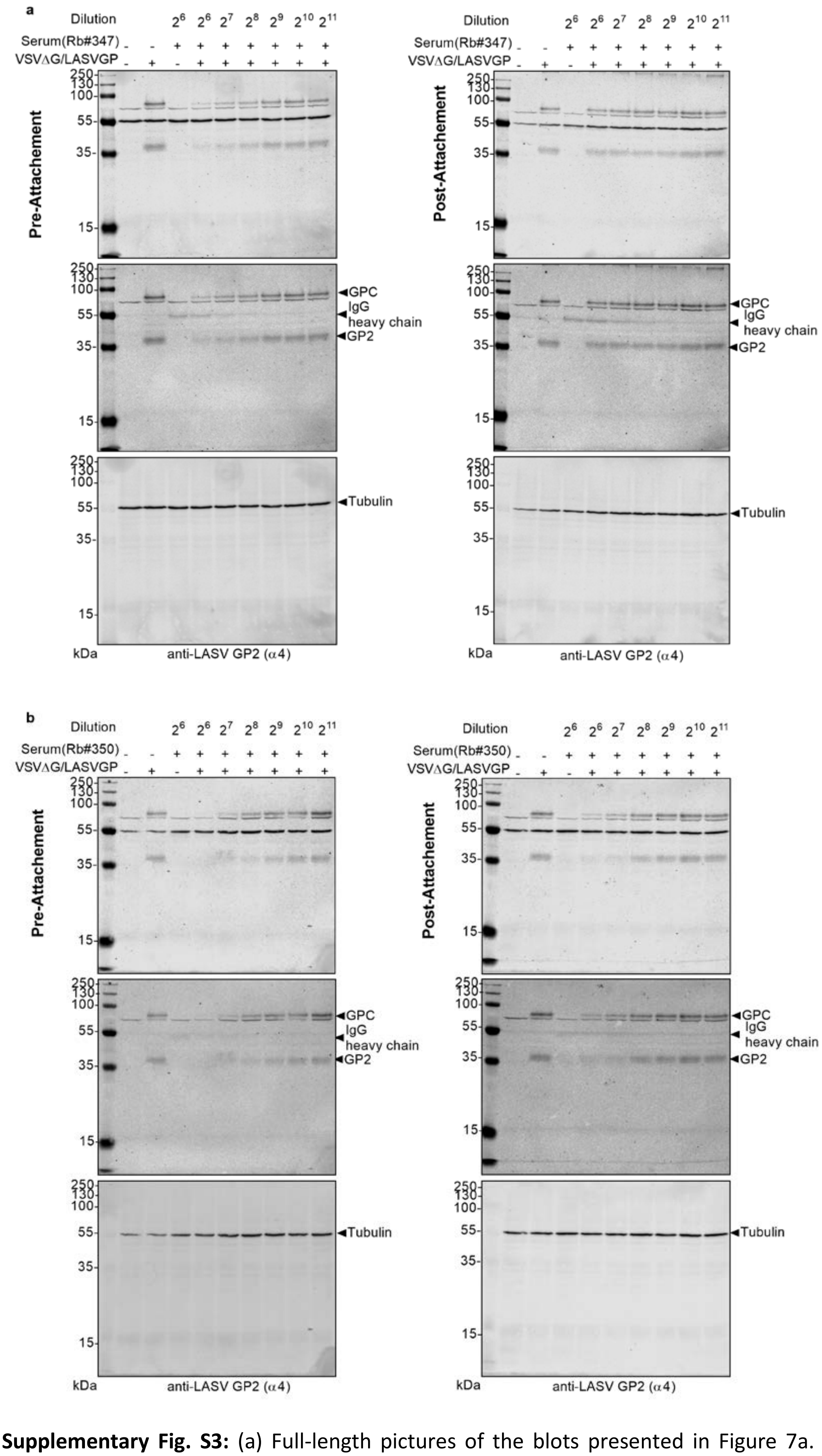


**Supplementary Fig. S3:** (a) Full-length pictures of the blots presented in Figure 7a. (b) Full-length pictures of the blots presented in Figure 7b.

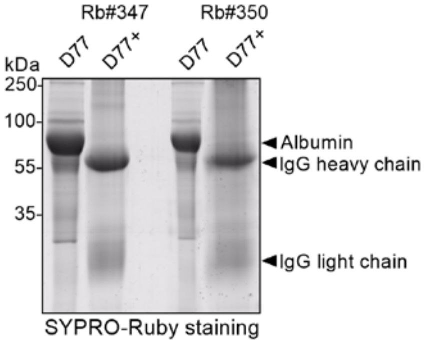


**Supplementary Fig. S4:** Picture of the gel presented in Figure 8a. The gel image was cropped after scanning and saved as original file. The full-length picture of the gel is no longer available.
