# Supplementary material for: Adjuvant formulated virus-like particles expressing native-like forms of the Lassa virus envelope surface glycoprotein are immunogenic and induce antibodies with broadly neutralizing activity
Source: NPJ Vaccines. 2020 Aug 4;5:71. doi: 10.1038/s41541-020-00219-x (PMC7403343; doi:10.1038/s41541-020-00219-x)
Supplement: Supplementary file 2 — Reporting Summary [file 41541_2020_219_MOESM2_ESM.pdf]

nature research

Corresponding author(s): Thomas Strecker

Last updated by author(s): Jul 7, 2020

Reporting Summary

Nature Research wishes to improve the reproducibility of the work that we publish. This form provides structure for consistency and transparency in reporting. For further information on Nature Research policies, see Authors & Referees and the Editorial Policy Checklist .

Statistics

For all statistical analyses, confirm that the following items are present in the figure legend, table legend, main text, or Methods section.

n/a	Confirmed	
☐	☒	The exact sample size (*n*) for each experimental group/condition, given as a discrete number and unit of measurement
☐	☒	A statement on whether measurements were taken from distinct samples or whether the same sample was measured repeatedly
☐	☒	The statistical test(s) used AND whether they are one- or two-sided<br>*Only common tests should be described solely by name; describe more complex techniques in the Methods section.*
☒	☐	A description of all covariates tested
☒	☐	A description of any assumptions or corrections, such as tests of normality and adjustment for multiple comparisons
☐	☒	A full description of the statistical parameters including central tendency (e.g. means) or other basic estimates (e.g. regression coefficient) AND variation (e.g. standard deviation) or associated estimates of uncertainty (e.g. confidence intervals)
☒	☐	For null hypothesis testing, the test statistic (e.g. *F*, *t*, *r*) with confidence intervals, effect sizes, degrees of freedom and *P* value noted<br>*Give P values as exact values whenever suitable.*
☒	☐	For Bayesian analysis, information on the choice of priors and Markov chain Monte Carlo settings
☒	☐	For hierarchical and complex designs, identification of the appropriate level for tests and full reporting of outcomes
☒	☐	Estimates of effect sizes (e.g. Cohen's *d*, Pearson's *r*), indicating how they were calculated

Our web collection on statistics for biologists contains articles on many of the points above.

Software and code

Policy information about availability of computer code

Data collection

Western blot analysis: Image Studio Lite Ver 3.1 (Odyssey Infrared Imaging System (LI-COR Biosciences);
Immunofluorescence imaging: AxioVision SE64 Rel. 4.9 (Zeiss observer 7 /ApoTome, BPM) and Leica Application Suite X (Confocal laser scanning microscopy – Leica SP5 II AOBS);
ELISA: Gen5 2.00 (Epoch Microplate Spectrophotometer, Biotek) and (Synergy H1 Multi-Mode Microplate Reader, Biotek)
Pseudotype-based virus neutralization assay: MARS software (FLUOstar Omega plate reader);
Flow cytometry-based virus neutralization assay: FlowJo v10.0 software (BD FACSCalibur);
Neutralization assay using authentic LASV: AID EliSpot Reader Version 7.0 (ELISpot reader);
Protein concentration measurement: CFR21 Software (NanoPhotometer® NP80, Implen)

Data analysis

GraphPad Prism 8, Fiji ImageJ software, FlowJo software v10.0

For manuscripts utilizing custom algorithms or software that are central to the research but not yet described in published literature, software must be made available to editors/reviewers. We strongly encourage code deposition in a community repository (e.g. GitHub). See the Nature Research guidelines for submitting code & software for further information.

Data

Policy information about availability of data

All manuscripts must include a data availability statement. This statement should provide the following information, where applicable:
- Accession codes, unique identifiers, or web links for publicly available datasets
- A list of figures that have associated raw data
- A description of any restrictions on data availability

The data reported in this paper are available from the corresponding author upon request.

October 2018

Field-specific reporting

Please select the one below that is the best fit for your research. If you are not sure, read the appropriate sections before making your selection.

☒ Life sciences ☐ Behavioural & social sciences ☐ Ecological, evolutionary & environmental sciences

For a reference copy of the document with all sections, see nature.com/documents/nr-reporting-summary-flat.pdf

Life sciences study design

All studies must disclose on these points even when the disclosure is negative.

Sample size	n=2; proof of concept
Data exclusions	No data were excludet
Replication	All attempts at replication were successful
Randomization	Not relevant for the study
Blinding	Not relevant for the study

Reporting for specific materials, systems and methods

We require information from authors about some types of materials, experimental systems and methods used in many studies. Here, indicate whether each material, system or method listed is relevant to your study. If you are not sure if a list item applies to your research, read the appropriate section before selecting a response.

Materials & experimental systems

n/a	Involved in the study	
☐	☒	Antibodies
☐	☒	Eukaryotic cell lines
☒	☐	Palaeontology
☐	☒	Animals and other organisms
☐	☒	Human research participants
☒	☐	Clinical data

Methods

n/a	Involved in the study	
☒	☐	ChIP-seq
☐	☒	Flow cytometry
☒	☐	MRI-based neuroimaging

Antibodies

Antibodies used

- Monoclonal mouse antibody AC1, specific for the GP1 (provided by M. C. Georges-Courbot, Unit of Biology of Viral Emerging Infections, Institute Pasteur, Lyon, France)
- Polyclonal antibodies raised in rabbits: α231 recognizing LASV GP1, α3 recognizing LASV GP2, and α4 detecting LASV GP2.
- Guinea pig anti-VSV polyclonal serum (provided by W. Garten (Institute of Virology, Marburg, Germany))
- Mouse monoclonal beta-tubulin antibody from SIGMA Life Science (T9026-.2ML; LOT:066M4870V).
- Human monoclonal LASV Glycoprotein Complex [Cl: 37.7H] (absolute antibody; LOT: T1836B44)
- Anti-rabbit secondary antibody labeled with Alexa Fluor 680 (Invitrogen, A32734)
- Anti-mouse secondary antibody labeled with Alexa Fluor Plus 800 (Invitrogen, A32789)
- Anti-rabbit secondary antibodies labeled with Alexa Fluor 568 (Invitrogen, A11011, LOT: 685221)
- Anti-rabbit secondary antibodies labeled with Alexa Fluor 488 (Invitrogen, A21441; LOT: 1796684)
- Anti-rabbit secondary antibody labeled with horseradish peroxidase (DAKO, P0448; LOT:20061231)
- Anti-guinea pig secondary antibody labeled with fluorescein isothiocyanate (DAKO, F0233; LOT: 010(031))
- Anti-human secondary antibody labeled with fluorescein isothiocyanate (DAKO, F0202; LOT: F020210055101)

Validation: Data provided in the manuscript

Eukaryotic cell lines

Policy information about cell lines

Cell line source(s):
African green monkey kidney cells (Vero E6, ATCC CRL-1586)
Human hepatoma cells (HuH7, ATCC CCL-185)

	Madin-Darby canine kidney strain II cells (MDCK II, ATCC CCL-34)<br>Human embryonic kidney 293T (HEK293T, ECACC 12022001)<br>Human monocytic cell line derived from an acute monocytic leukemia patient (THP-1 cells, ATCC-TIB-202)<br>Baby hamster kidney cells (BHK; CVCL_1915)
Authentication	Cell lines were authencicated by DNA sequencing and Short Tandem Repeat (STR) profiling.
Mycoplasma contamination	All cell lines were tested negative for mycoplasma contamination.
Commonly misidentified lines<br>(See ICLAC register)	No misidentified cell lines were used in this study.

Animals and other organisms

Policy information about studies involving animals; ARRIVE guidelines recommended for reporting animal research

Laboratory animals	New Zealand White rabbits (Oryctolagus cuniculus)
Wild animals	The study did not involve wild animals.
Field-collected samples	The study did not involve samples collected from the field.
Ethics oversight	see above

Note that full information on the approval of the study protocol must also be provided in the manuscript.

Human research participants

Policy information about studies involving human research participants

Population characteristics	see above
Recruitment	I) Human sera were collected in a highly LASV-endemic area of Guinea in 2014 (Fichet-Calvet and Magassouba, unpublished data).<br>II) European human serum samples from healthy anonymous donors
Ethics oversight	I) Approval for the investigation was obtained from the National Ethics Commission of Guinea (permit no. 12/CNERS/12).

Note that full information on the approval of the study protocol must also be provided in the manuscript.

Flow Cytometry

Plots

Confirm that:

- [x] The axis labels state the marker and fluorochrome used (e.g. CD4-FITC).
- [x] The axis scales are clearly visible. Include numbers along axes only for bottom left plot of group (a 'group' is an analysis of identical markers).
- [x] All plots are contour plots with outliers or pseudocolor plots.
- [x] A numerical value for number of cells or percentage (with statistics) is provided.

Methodology

Sample preparation	Cells origin specified under " Eukaryotic cell lines". For flow cytometric analysis cells were seeded in 6-well plates one day before performing the experiment. The virus-antibody mixture was added to the cell monolayers for 22 h. Cells were gently harvested by trypsinization, fixed with 4% PFA for 20 min at RT, and permeabilized with saponin-buffer (PBS, 3% FBS, 2mM EDTA, 0.001% sodium azide, 0.5% saponin) for 30 min at RT. Samples were incubated with polyclonal guinea pig-αVSV serum at dilution 1:1000, followed by rabbit α-guinea pig FITC-conjugated antibody at dilution 1:1000 for 1 hour at 4°C each. After each incubation step, cells were washed (PBS, 3% FBS, 2mM EDTA, 0.001% sodium azide) twice and finally subjected to flow cytometry analysis.
Instrument	BD FACSCalibur
Software	Data were collected with Cell Quest Pro V5.2, FlowJo v10.0 software was used for data analysis.
Cell population abundance	Data were obtained from established cell lines of single cell type, no sorting or specific purity controls were used. Since cell lines used in the experiments differ in cell size and granularity, specific instrument stetting were used for each cell line.
Gating strategy	For all experiments living cells were distinguished from cell fragments by FSC-H/SSC-H, referring to cell size and granularity in the starting population. Since established cell lines were used in the experiments only a single cell population was expected. Based on cell population considered as "living" further analyses for VSVdG/LASVGP infection were performed. For each cell line

untreated VSVdG/LASVGP-infected cells were used as positive controls. Uninfected cells treated with highest concentration of each serum was used as negative controls.

☐ Tick this box to confirm that a figure exemplifying the gating strategy is provided in the Supplementary Information.
